# *Nitrospina*-Like Bacteria Are Potential Mercury Methylators in the Mesopelagic Zone in the East China Sea

**DOI:** 10.3389/fmicb.2020.01369

**Published:** 2020-07-03

**Authors:** Yuya Tada, Kohji Marumoto, Akinori Takeuchi

**Affiliations:** ^1^Department of Environment and Public Health, National Institute for Minamata Disease, Kumamoto, Japan; ^2^Center for Environmental Measurement and Analysis, National Institute for Environmental Studies, Ibaraki, Japan

**Keywords:** mercury, methylmercury, marine bacteria, *hgcAB* genes, 16S rRNA gene, functional module

## Abstract

In natural environments, the production of neurotoxic and bioaccumulative methylmercury (MeHg) is mediated by microorganisms carrying the genes *hgcA* and *hgcB*. However, the contribution of these microorganisms to mercury (Hg) methylation or MeHg accumulation in the ocean is poorly understood. Here we determined the total Hg (THg) and MeHg concentrations in seawater samples and conducted a metagenomic survey of the *hgcAB* genes and functional modules involved in metabolic pathways in the East China Sea (ECS). In the metagenomic analyses, we used paired-end reads and assembled contigs for *hgcAB* enumeration and phylogenetic analyses in the seawater column. To evaluate the relative abundance of *hgcAB* in the metagenomic data, we estimated the abundance of *recA* (single-copy gene of bacteria) as well and then compared them. Moreover, the profiles of prokaryotic community composition were analyzed by 16S rRNA gene (V4 region) deep-sequencing. In the mesopelagic layers, the *hgcA* sequences were detected, and there was a positive correlation between *hgcA* abundance relative to the *recA* and MeHg concentrations. Thus, the quantification of the *hgcA* sequences could provide valuable information to evaluate the potential environments of microbial MeHg accumulation in the seawater column. A phylogenetic analysis using the assembled contigs revealed that all of the *hgcA* sequences in the mesopelagic layers were affiliated with *Nitrospina*-like sequences. The 16S rRNA gene analysis revealed that Nitrospinae were abundant in the mesopelagic layers. Although the lineages of Deltaproteobacteria, Firmicutes, and Spirochaetes were detected in the seawater column, their *hgcAB* sequences were not detected in our metagenomes, despite the fact that they are closely related to previously identified Hg methylators. The metabolic pathway analysis revealed that the modules related to sulfur and methane metabolism were prominent in the mesopelagic layers. However, no *hgcA* sequences affiliated with sulfate-reducing bacteria (SRB) or methanogens were detected in these layers, suggesting that these bacteria could not be strongly involved in the Hg accumulation in the seawater column. Our results indicate that *Nitrospina*-like bacteria with *hgcAB* genes could play a critical role in microbial Hg accumulation in the oxygenated mesopelagic layers of the ECS.

## Introduction

Consumption of marine fish is considered as a major route whereby humans and marine mammals can become exposed to toxic and bioaccumulative methylmercury (MeHg) (Food and Agriculture Organization [FAO], 2013). The bioaccumulation of mercury (Hg) in fish depends on the concentration of MeHg rather than the total Hg (THg) concentrations in freshwater and seawater columns (Boudou and Ribeyre, 1997). Information regarding the production and the distribution of MeHg in marine environments is critical for understanding the Hg cycle in marine food webs, and previous studies have shown that MeHg production in marine environments mainly occurs in the sediments through the biologically mediated conversion of Hg(II) by anaerobic microorganisms (Boudou and Ribeyre, 1997; Hammerschmidt and Fitzgerald, 2004; Sunderland et al., 2004; Eckley and Hintelmann, 2006; Kerin et al., 2006). In contrast, the spatial distributions of THg and MeHg concentrations in the open ocean indicated that active Hg methylation or MeHg accumulation probably occurs in the coastal and pelagic seawater column (Monperrus et al., 2007; St. Louis et al., 2007; Kirk et al., 2008; Sunderland et al., 2009; Blum et al., 2013; Kim et al., 2017). In addition, incubation experiments have demonstrated that *in situ* MeHg production can occur in oxygenated marine environments (Monperrus et al., 2007; Lehnherr et al., 2011). These results indicate that MeHg entry into marine food webs in the open ocean depends on *in situ* production in the seawater column rather than being linked to sediment MeHg (Kraepiel et al., 2003). Thus, MeHg formation in the seawater column is hypothesized to be a key process for MeHg bioaccumulation in marine environments.

In terms of the oceanic distribution of MeHg, higher concentrations of MeHg have been consistently observed in the mesopelagic layers (depths of ca. 200–1000 m), including the oxygen minimum zone (Sunderland et al., 2009; Munson et al., 2015; Kim et al., 2017), than in the surface layers and the deeper waters. Although the presence of MeHg in these layers is assumed to be attributable to MeHg production from the microbial remineralization of organic matter (Mason and Fitzgerald, 1993; Laurier et al., 2004; Cossa et al., 2009; Sunderland et al., 2009; Hammerschmidt and Bowman, 2012), there is limited information regarding which phylogenetic lineages of microorganisms contribute to MeHg production in the seawater column.

Microbial Hg methylation has been previously confirmed using specific anaerobic strains of sulfate-reducing bacteria (SRB) (Compeau and Bartha, 1985; Gilmour and Henry, 1991; Kerry et al., 1991; Gilmour et al., 1992) and iron-reducing bacteria (IRB) (Yu et al., 2012), and methanogens (Hamelin et al., 2011). Although it has been documented that these anaerobic microorganisms are responsible for Hg methylation and MeHg accumulation in the oxygen-deficient environments that characterize sediments and soils (Benoit et al., 2003), a microbial community analysis using the 16S rRNA gene failed to identify Hg-methylating bacteria to be affiliated with SRB and IRB in the pelagic seawater column where MeHg production was observed (Malcolm et al., 2010).

A comparative genomic analysis of confirmed Hg methylators, including SRB and IRB, revealed the key genes (*hgcA* and *hgcB*) essential for Hg methylation (Parks et al., 2013). The *hgcA* gene encodes a corrinoid-dependent protein that presumably functions as part of a methyltransferase similar to the corrinoid iron–sulfur protein of the reductive acetyl-coenzyme A (CoA) pathway. The *hgcB* gene encodes an associated ferredoxin protein that potentially reduces the corrinoid center of the HgcA protein. Both genes are globally prevalent and widely distributed in aquatic environments, including marine water columns, sediments, and sea ice (Podar et al., 2015; Gionfriddo et al., 2016).

Several studies have detected *hgcA* sequences in all investigated marine sediments, but these are rarely found in marine water columns (Podar et al., 2015; Munson et al., 2018). These findings thus raise questions regarding the magnitude and the extent of microbial MeHg accumulation at the boundary of coastal and open ocean environments. Several studies have suggested that MeHg in the coastal seawater is derived from ocean floor sediments (Hammerschmidt and Fitzgerald, 2006; Hollweg et al., 2010). In the present study, we addressed these questions through surveying areas in the East China Sea (ECS), one of the largest marginal seas in the western North Pacific, a large part (approximately 80%) of which overlies the continental shelf. In this area, the Kuroshio Current flows consistently from southwest to northeast, and it is important for the oceanic circulation of nutrients and other materials at the regional and global scales (e.g., Chen and Wang, 1999; Guo et al., 2012). These geophysical features are assumed to affect the Hg and MeHg distribution in the seawater column (Hammerschmidt and Fitzgerald, 2006; Kim et al., 2017). Thus, in the present study, we aimed to examine the depth distribution of the *hgcA*, *hgcB*, and 16S rRNA genes and the functional modules associated with the relevant metabolic pathways, as well as the concentrations of THg and MeHg in the seawater column, and to determine which phylogenetic lineages of microorganisms are involved in the accumulation of MeHg in the ECS.

## Materials and Methods

### Seawater Sampling and Hydrological Conditions

**FIGURE 1:**
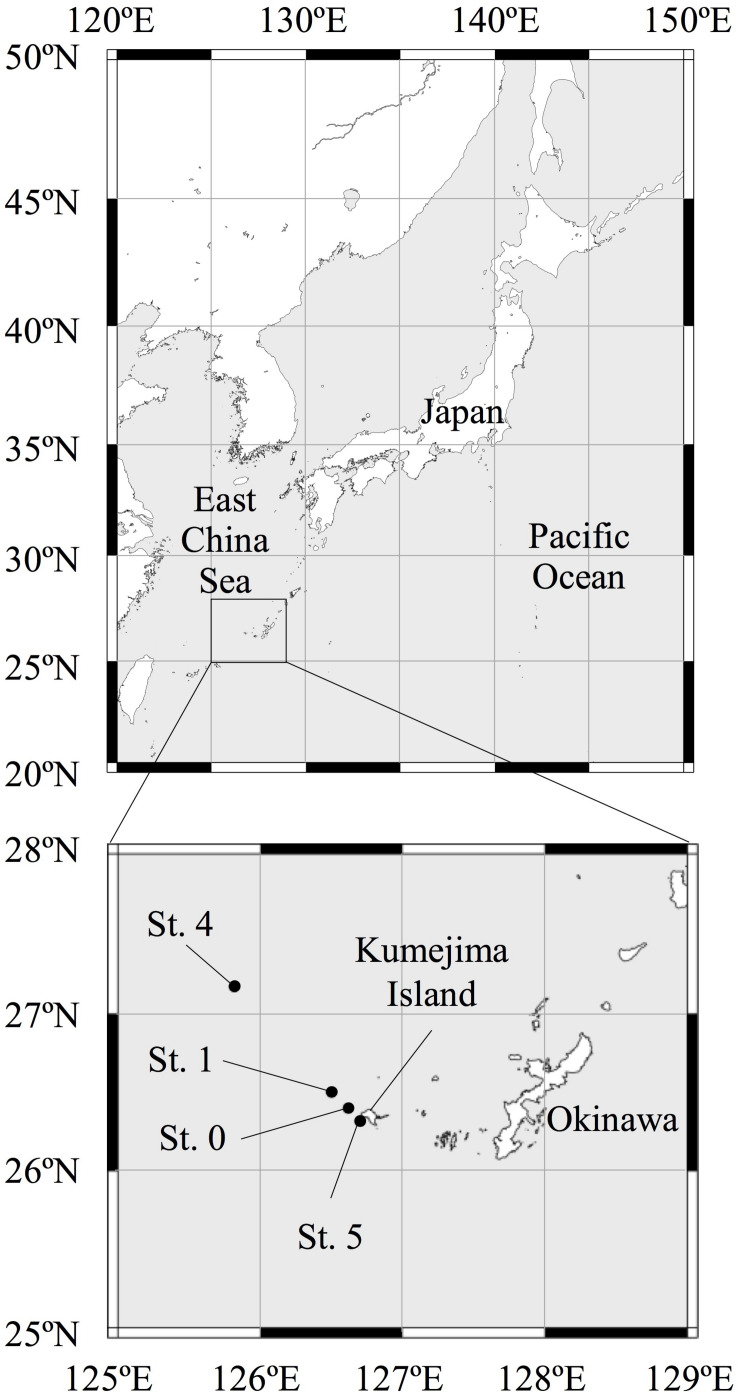
Map of the sampling stations in the East China Sea.

For the purposes of this study, we collected seawater from four sampling stations (Sts. 0, 1, 4, and 5) in the ECS (Figure 1). We collected multiple seawater samples from 0- to 800-m depth at Sts. 0 and 1 and from 0- to 200-m depth at Sts. 4 and 5 around Kumejima Island in the ECS from 13 to 16 March 2018 (Table 1 and Supplementary Table S1). Station 4 is located on the continental shelf of the ECS. We could not collect the bottom water from Sts. 0 and 1 due to the limited length of our CTD wire and the bad weather.

**TABLE 1:** Station names, sampling date, position, and maximum depth at sampling stations in the East China Sea.

Station name	Sampling date	Latitude	Longitude	Maximum depth (m)
St. 0	14 Mar 2018	26° 24′N	126° 37′E	1700
St. 1	13 Mar 2018	26° 30′N	126° 30′E	1800
St. 4	15 Mar 2018	27° 10′N	125° 49′E	238
St. 5	16 Mar 2018	26° 19′N	126° 42′E	297

Seawater samples were collected using an external spring Niskin seawater sampler [cleaned with 1 M HNO_3_ (Poisonous Metal Analysis grade, Kanto Chemical Co.)] with Teflon-coated inner wall and 1-L PFA bottles (cleaned with 1 M HNO_3_). To determine the THg and MeHg concentrations, approximately 1 L of seawater was collected in the PFA bottles. Of the 1-L seawater samples collected, 200 mL was subsampled into 250-mL PFA bottles (cleaned with acid) and preserved for the THg analysis by adding 1.0 mL of concentrated ultrapure HCl (Kanto Chemical Co.) and 1.0 mL of BrCl (Guaranteed Reagent; Kanto Chemical Co.) (0.2 mol L^–1^). The remaining 800 mL, which was used for the MeHg analysis, was preserved by adding 4.0 mL of 10 M ultrapure H_2_SO_4_ (TAMAPURE-AA-100; Tama Chemicals Co.). The treated water samples were stored at 4°C in the dark until their use for further analysis. The absence of Hg contamination from the Niskin sampler was confirmed by determining the Hg concentrations in the filled ultrapure water in the lab before the oceanic investigation.

For the metagenomic analysis, we collected 10 L of seawater samples in a sterilized 10-L plastic container at 0- [the subsurface chlorophyll maximum (SCM)], 100-, 200-, 500-, and 800-m depth at Sts. 0 and 1 and at 0 (SCM), 100, and 200 m at Sts. 4 and 5. These samples were stored in the dark at approximately 10°C and filtered using a 0.22-μm-pore-size Sterivex-GP (polyethersulfone membrane) cartridge filter (Millipore, MA, United States) within 6 h after collection (within 12 h for the St. 4 samples), and the prokaryotic cells were collected. These cartridge filters were stored at −80°C until their use for further analysis.

The macronutrient samples were collected in 10-mL acrylic tubes and stored at −30°C until their use for further analysis. Nitrate plus nitrite (NO_3_ + NO_2_), phosphate (PO_4_), and silicic acid [Si(OH)_4_] concentrations were determined using a QuAAtro39 segmented continuous flow analyzer (Bran + Luebbe). The particulate organic carbon (POC) and the particulate nitrogen (PN) in the seawater were filtered onto pre-combusted (450°C for 4 h) GF/F filters (25 mm in diameter, Whatman). These filters were stored at −30°C until their use for further analysis. Prior to the analysis, the filters were treated with HCl (Guaranteed reagent; Kanto Chemical Co.) fumes in a container for 24 h to remove inorganic carbon (Hedges and Stern, 1984). The concentrations of POC and PN were measured using a FLASH EA1112 elemental analyzer (Thermo Finnigan).

The physicochemical parameters of seawater, including water temperature, salinity, and chlorophyll *a* (Chl. *a*) and dissolved oxygen (DO) concentrations, were determined using a CTD system (RINKO-Profiler; JFE Advantec Co. Ltd.). We determined the depth of the SCM layers based on the CTD profiles.

### THg and MeHg Analyses

Analyses of dissolved THg and MeHg were performed according to the protocols described by Marumoto et al. (2018). Briefly, THg was measured using EPA Method 1631 (U. S. Environmental Protection Agency, 2002). The concentration of THg in each sample was determined using cold vapor atomic fluorescence spectrometry and gold amalgamation (RA-FG^+^; Nippon Instruments Corporation) after the generation of Hg (0) using 1 mL of 20% (w/v) SnCl_2_ as a reducing agent. The precision of the THg analysis was verified using multiple measurements of BCR579 standard reference material (certified range of 1900 ± 500 pg L^–1^) (9.5 ± 2.5 pM). We found that the determined values (1850 ± 60 pg L^–1^, *n* = 6; 9.3 ± 0.3 pM) were invariably within this range. The method detection limit (MDL) value was calculated using method blank solutions with ultrapure water and reagents. The MDL for THg was 49 pg L^–1^ (0.25 pM; *n* = 12), defined as thrice the standard deviation of the blank. Multiple measurements of the blank (ultrapure water with reagents) showed that the THg concentration was 46 ± 16 pg L^–1^ (0.23 ± 0.08 pM; *n* = 12). The instrument blank value was determined with a blank trap column (less than 1 pg). The blank value of the reagents (BrCl solution) was 3.4 pg mL^–1^ (0.017 nM), calculated by determining the increments of THg concentrations in blanks with different amounts of reagents added. The THg concentrations of seawater samples were calculated by subtracting the reagent and the instrument blank values.

The analytical procedure used to determine MeHg was based on solvent extraction with dithizone-toluene and Na_2_S solutions (Ministry of the Environment, 2004). The MeHg concentrations in the Na_2_S solutions were determined by ethylation using NaB(C_2_H_5_)_4_, preconcentration onto a Tenax trap, thermal desorption, and gas chromatography with atomic fluorescence detection, based on the methods described by Logar et al. (2002). The MDL (calculation process was the same as THg) for MeHg was 1.4 pg L^–1^ (0.007 pM), when the blank solutions (*n* = 8) using ultrapure water were measured. The blank value of MeHg using ultrapure water was 0.85 ± 0.46 pg L^–1^ (0.004 ± 0.002 pM) (*n* = 3). In addition, the recovery of MeHg was 99 ± 3% (*n* = 7) based on the recovery of a spike of a known concentration of MeHg obtained from the alkaline dissolution of DORM-2 (certified range: 4.47 ± 0.32 mg kg^–1^ as dry weight), which is an international standard reference material for MeHg in dogfish. At least one DORM-2 solution was measured in every five MeHg sample measurements.

### Prokaryotic Cell Abundance

To measure prokaryotic cell abundance, 10-mL seawater samples were collected in sterile polypropylene tubes (15 mL, Nalgene) and fixed with paraformaldehyde (final concentration, 2%). After filtration using 0.2-μm-pore-size polycarbonate membrane filters (GTTP02500, 25 mm in diameter, Millipore), the retained prokaryotic cells were stained with SYBR Gold (final concentration, 2.5 × 10^–4^) for 10 min in the dark at room temperature (Chen et al., 2001) and counted under an epifluorescence microscope.

### DNA Extraction

DNA was extracted from Sterivex-GP filters using an enzyme/phenol-chloroform protocol (Boström et al., 2004; Tada and Suzuki, 2016). The bacterial cells were lyzed *via* lysozyme digestion for 60 min (final concentration, 1 mg mL^–1^) at 37°C and an overnight digestion at 55°C with proteinase K (final concentration, 0.2 mg mL^–1^) and sodium dodecyl sulfate [final concentration, 1% (vol/vol)]. The lysate was extracted twice with an equal volume of phenol–chloroform–isoamyl alcohol (25:24:1) and once with an equal volume of chloroform–isoamyl alcohol (24:1); the aqueous layer obtained was transferred to a sterilized tube. DNA was precipitated with isopropanol and ammonium acetate (final concentration, 0.3 M). The DNA solutions were treated with RNase (final concentration, 0.1 μg mL^–1^) for 15 min at room temperature. The extracted DNA was stored at −80°C until further analysis. The quality of the metagenomic DNA was assessed by 1% agarose gel electrophoresis.

### 16S rRNA Gene Deep-Sequencing Analysis

The bacterial and archaeal 16S rRNA (V4 region) gene fragments were amplified using the following primers, with adaptor sequences from Illumina (San Diego, CA, United States): 515F, ACACTCTTTCCCTACACGACGCTCTTCCGATCT-GTGCCAGCMGCCGCGGTAA; and 806RB, GTGACTGGAGTTCAGACGTGTGCTCTTCCGATCT-GGACTACNVGGGTWTCTAAT (Caporaso et al., 2011; Apprill et al., 2015). The PCR program included an initial denaturation step for 5 min at 94°C, followed by 25 cycles of denaturation (94°C, 30 s), annealing (50°C, 30 s), and extension (72°C, 30 s); a final extension for 3 min at 72°C completed the amplification reaction. The amplicons were visualized by electrophoresis on SYBR Gold-stained 1.5% agarose gels. These PCR amplicons were then sequenced considering 2× 250-bp paired-end sequences on the Illumina MiSeq platform. The raw 16S rRNA sequence data have been deposited in the DNA Data Bank of Japan-Sequence Read Archive (DDBJ-SRA) under accession number DRA009218.

Quality filtering for noise and short read sequences removal was completed in the QIIME pipeline^1^. The chimeras were removed with USEARCH (Edgar et al., 2011), using the Greengenes16S rRNA gene dataset (McDonald et al., 2012) as reference. The remaining high-quality sequences were clustered into operational taxonomic units (OTUs) using a 97% similarity threshold.

### Metagenome Sequencing

The metagenomic DNA (50–100 ng total DNA) was barcoded by sample and used for library preparation. The paired-end libraries were generated with an insert size of ca. 350 bp, using a NEBNext Ultra DNA Library Prep kit following the manufacturer’s protocols (New England Bio Labs). Sequencing was performed by Novogene Bioinformatics Technology (Beijing, China), using Illumina HiSeq 2500 and 2× 250-bp paired-end sequencing. The raw sequence data for the metagenomes have been deposited in the DDBJ-SRA (accession no.: DRA008415).

### Metagenomic Sequence Data Analyses

A flowchart of the metagenomic sequence analyses conducted using paired-end reads and assembled contigs is presented in Supplementary Figure S1. For counting the *hgcA* and *hgcB* genes in the paired-end reads obtained from the metagenomic sequencing data, the Illumina adaptor sequences were first removed using Cutadapt (Martin, 2011). After the paired-end assembly with Paired-End reAd mergeR (Zhang et al., 2013), low-quality sequences (Q-value, <20; sequence length, <300 bp) were trimmed using FastXToolKit^2^ and PRINSEQ (Schmieder and Edwards, 2011). The genes in the metagenomic sequences were predicted using MetaGeneAnnotator (Noguchi et al., 2008).

After the gene prediction, putative open reading frame reads were extracted and translated into amino acid sequences using a bacterial translation table (NCBI transl_table = 11) and a Perl script. For the detection of *hgcA* and *hgcB* sequences in the metagenomes, a hidden Markov model (HMM) profile was constructed using HMMER v3.2.1 (Eddy, 2009), with an e-value cutoff of 10^–5^. To develop the HMM profiles for *hgcA* and *hgcB*, 145 and 128 representative sequences (Gilmour et al., 2013, 2018; Gionfriddo et al., 2016; Christensen et al., 2019; Jones et al., 2019) (Supplementary Tables S2, S3) were aligned using MEGA-X software (Kumar et al., 2018). To re-check the specificity of HMMs for *hgcAB* sequences, we conducted a local search using hmmsearch (HMMER v3.2.1) and the *hgcAB* reference sequences described above. For counting the *hgcAB* genes, the *hgcA*-like sequences without a conserved cysteine C93 motif, as a predicted cap helix (Parks et al., 2013; Smith et al., 2015; Gionfriddo et al., 2016), were trimmed. For *hgcB*, the sequences without two strictly conserved CX_2_CX_2_CX_3_C motifs were removed (Smith et al., 2015; Gionfriddo et al., 2016) after alignment using MEGA-X software. An effective estimation of *hgcAB* gene abundance from metagenomic sequences was normalized to the *recA* gene, which is an essential single-copy gene of bacteria (Tang et al., 2018). The HMM reference for the *recA* gene was constructed using PF00154 representative protein sequences.

For the phylogenetic analyses of *hgcA* and *hgcB* genes, the contigs with long metagenomic reads were assembled on MEGAHIT (Li et al., 2016) with default parameters (k-min = 21, k-max = 141, k-step = 12, *t* = 20) after removing the Illumina adaptors. The coverage of mapped reads for contig sequences was calculated using Bowtie2 (Langmead and Salzberg, 2012). After the construction of the contigs, gene prediction and *hgcAB* gene search were performed using the methods described above. Prior to the phylogenetic analysis, *hgcA* and *hgcB* sequences with >50% gaps (175 and 50 amino acids, respectively) were removed. In addition, *hgcA*- and *hgcB*-like sequences without the conserved motifs were trimmed (as described above). The phylogeny of *hgcAB* was analyzed using a maximum likelihood method in MEGA-X with bootstrap analysis (100 re-assemblages).

To evaluate the abundance of prokaryotic functional modules in the paired-end reads from the metagenomic sequencing data, we used the metabolic and physiological potential evaluator (MAPLE-2.3.1^3^; Takami et al., 2016) and the genes and module database (version 13 July 2018) defined by the Kyoto Encyclopedia of Genes and Genomes (KEGG) database. This software automatically maps the genes in the metagenomic sequences to the 796 functional modules in the KEGG database and estimates the module abundance and the module completion ratio (MCR) for each functional module.

### Statistical Analyses

The data were analyzed with R software v.3.4.3 (Pinheiro et al., 2017), using Spearman’s rank correlation to investigate the relationship between *hgcAB* abundance and Hg (THg and MeHg) concentrations. In the statistical analysis, values under the detection limit (represented by “not detected” in Supplementary Table S1) were treated as zero. A heatmap of functional module abundance was constructed using R software after standardization.

## Results

### Environmental Characteristics

**FIGURE 2:**
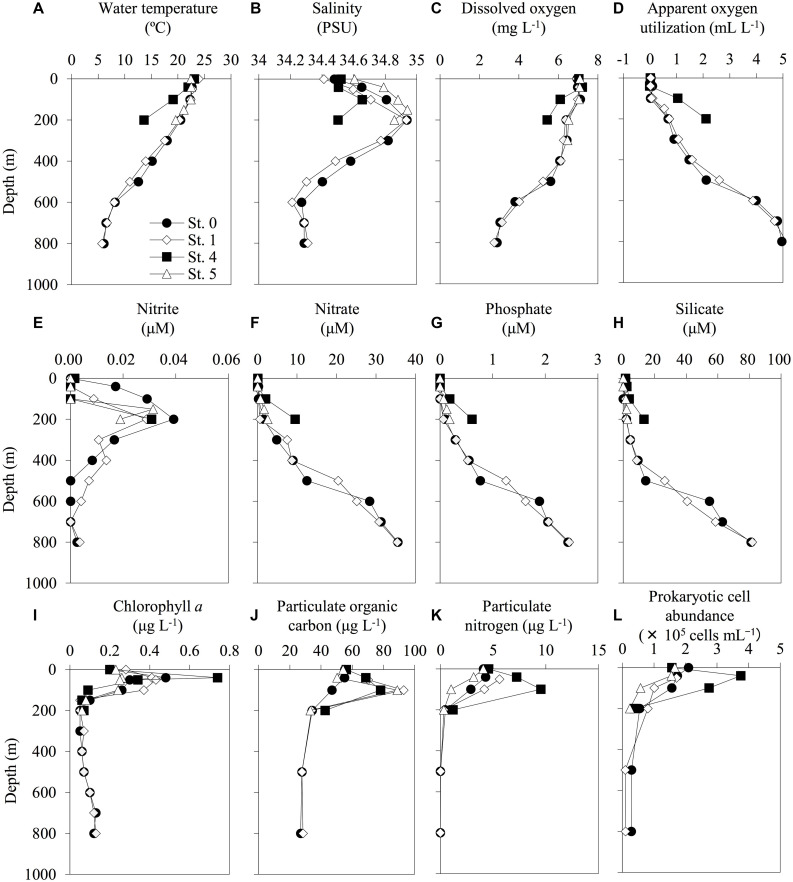
Depth profiles of the environmental factors at each sampling station. The profiles of water temperature **(A)**, salinity **(B)**, dissolved oxygen **(C)**, apparent oxygen utilization **(D)**, nitrite **(E)**, nitrate **(F)**, phosphate **(G)**, silicate **(H)**, chlorophyll *a* **(I)**, particulate organic carbon **(J)**, particulate nitrogen **(K)**, and prokaryotic cell abundance **(L)** were shown. The profiles do not extend to bottom waters (reference bottom depths in Table 1).

According to the CTD data, the seawater temperature and the DO concentrations decreased with increasing depth (Figure 2). Anaerobic conditions were not observed throughout the investigated seawater columns, and there was an enrichment of macronutrients (nitrate, phosphate, and silicate) in the mesopelagic layers. Compared with the other stations from which water samples were collected, the waters at St. 4 (above the continental shelf) had different hydrographic characteristics. For example, there were higher decreases in the gradients of both seawater temperature and DO concentration and increasing gradients of macronutrients with depth at St. 4 than at the other stations (Figure 2). Moreover, the highest levels of Chl. *a*, PN, and prokaryotic cell abundance were found at St. 4. The raw data for all of the environmental factors measured in this study are shown in Supplementary Table S1.

### Vertical Profiles of THg and MeHg

**FIGURE 3:**
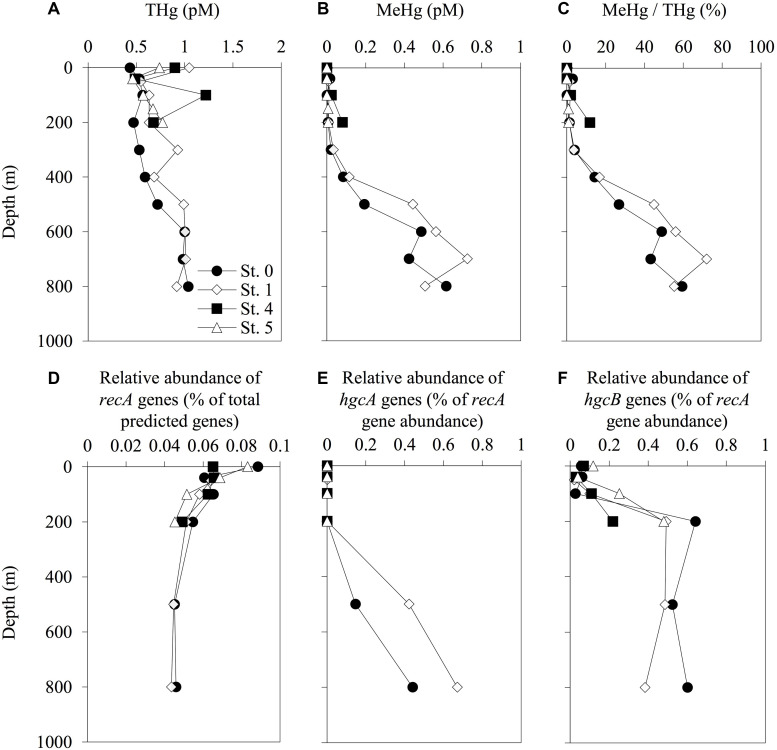
Depth profiles of total mercury (THg) **(A)**, methylmercury (MeHg) **(B)**, proportion of THg as MeHg **(C)**, relative abundance of *recA* genes among the total predicted genes **(D)**, and abundance of *hgcA* **(E)** and *hgcB* **(F)** sequences normalized to *recA* gene abundance in each sampling station. The abundance of *recA* and *hgcAB* genes was determined using the HMMER search on the paired-end reads from the metagenomic sequencing data. Reference libraries were constructed using PFam00154 for *recA*, and confirmed and predicted *hgcAB* sequences have been described in Supplementary Tables S2, S3. The profiles do not extend to the bottom waters.

The concentrations of THg in the seawater samples ranged from 0.43 pM (0-m depth at St. 0) to 1.22 pM (100-m depth at St. 4) (Figure 3). The THg concentrations at St. 0 gradually increased with depth from 0.43 pM at the surface to 1.04 pM at 800-m depth. No similar gradual increasing trend was observed at any of the other stations. We also observed increases in the concentrations of MeHg and in the proportions of MeHg to THg with depth at deeper stations (Sts. 0 and 1), with concentrations reaching 0.62 pM (59% of THg) at a depth of 800 m at St. 0 and 0.73 pM (72% of THg) at a depth of 700 m at St. 1. In contrast, the MeHg concentrations in the shallow layers (<200 m) were low (<0.1 pM) at all the stations, even at the shallow stations (Figure 3).

### Taxonomic Profiles

**FIGURE 4:**
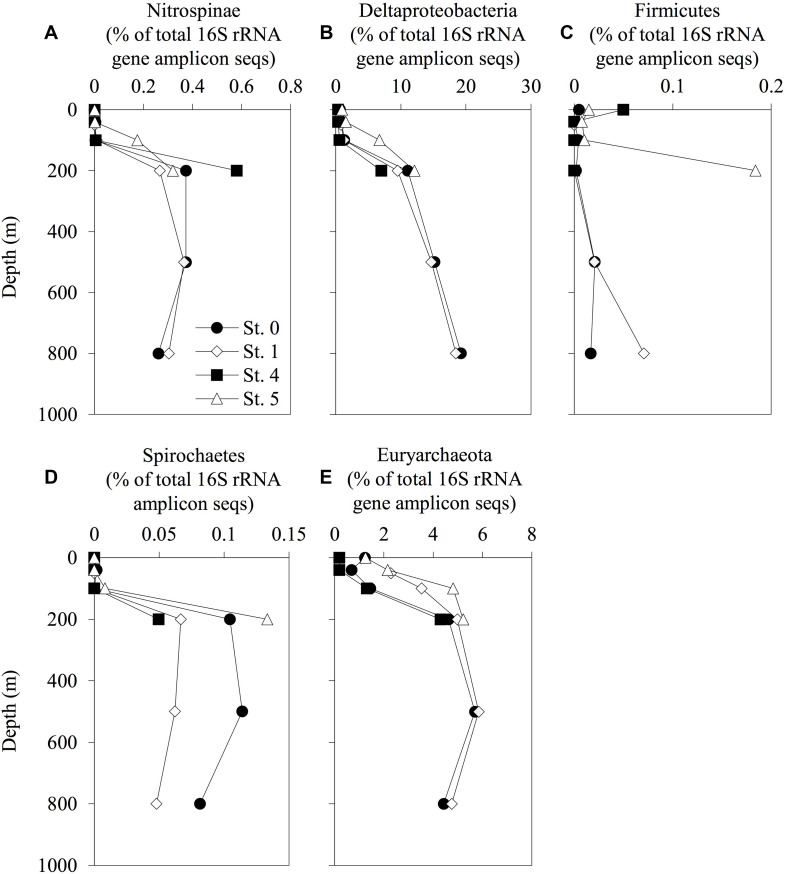
Depth distribution of prokaryotic phylotypes involved in mercury methylation. The relative abundance of Nitrospinae **(A)**, Deltaproteobacteria **(B)**, Firmicutes **(C)**, Spirochaetes **(D)**, and Euryarchaeota **(E)** and their total 16S rRNA amplicon sequences are displayed. The profiles do not extend to the bottom waters.

The vertical profiles of the phylogenetic lineages analyzed with 16S rRNA deep-sequencing, Nitrospinae, Deltaproteobacteria, Firmicutes, Spirochaetes, and Euryarchaeota (these lineages including some Hg methylators, or *hgcAB* carriers), are shown in Figure 4. The phylogenetic compositions and the vertical distributions of the other lineages are available in Supplementary Table S4 and Supplementary Figures S2–S4. The relative abundance of Nitrospinae, Deltaproteobacteria, Spirochaetes, and Euryarchaeota increased with depth. However, these trends were not observed in *Desulfobulbaceae*, *Desulfuromonadaceae*, and *Bdellovibrionaceae*, including some deltaproteobacterial Hg methylators or *hgcAB* carriers (Supplementary Figure S4). The proportion of Nitrospinae accounted for up to 0.37% of the total sequences at 500-m depth in Sts. 0 and 1. The relative abundance of Euryarchaeota increased at the same depth and accounted for 5.7 and 5.8% of the total sequences in Sts. 0 and 1, respectively. The proportion of Nitrospinae increased in the deep layers at St. 4, accounting for up to 0.58% of the total sequences, respectively. *Dehalococcoidales* (affiliated with Chloroflexi) and *Methanocellales* (affiliated with Euryarchaeota) were not detected in the 16S rRNA amplicon sequences obtained in the present study.

### Abundance and Distribution of *hgcAB* Genes in the Seawater Column

Our genetic analyses indicated that the relative abundance of *hgcA* sequences increased with depth, with the maximum values of 0.44 and 0.67% of *recA* gene abundance recorded at 800-m depth in Sts. 0 and 1, respectively (Figure 3 and Supplementary Table S5). We also found that the *hgcB* sequences were abundant in the layers below depths of 200 m, with relative abundances ranging from 0.02 to 0.64% of *recA* gene abundance. The *hgcB* sequences were also detected in the water column in Sts. 4 and 5 and increased with depth (0.22 and 0.48% of *recA* gene abundance at 200-m depth, respectively). However, these trends were not observed in the profiles of *hgcA*.

### Phylogenies of the *hgcAB* Genes

**FIGURE 5:**
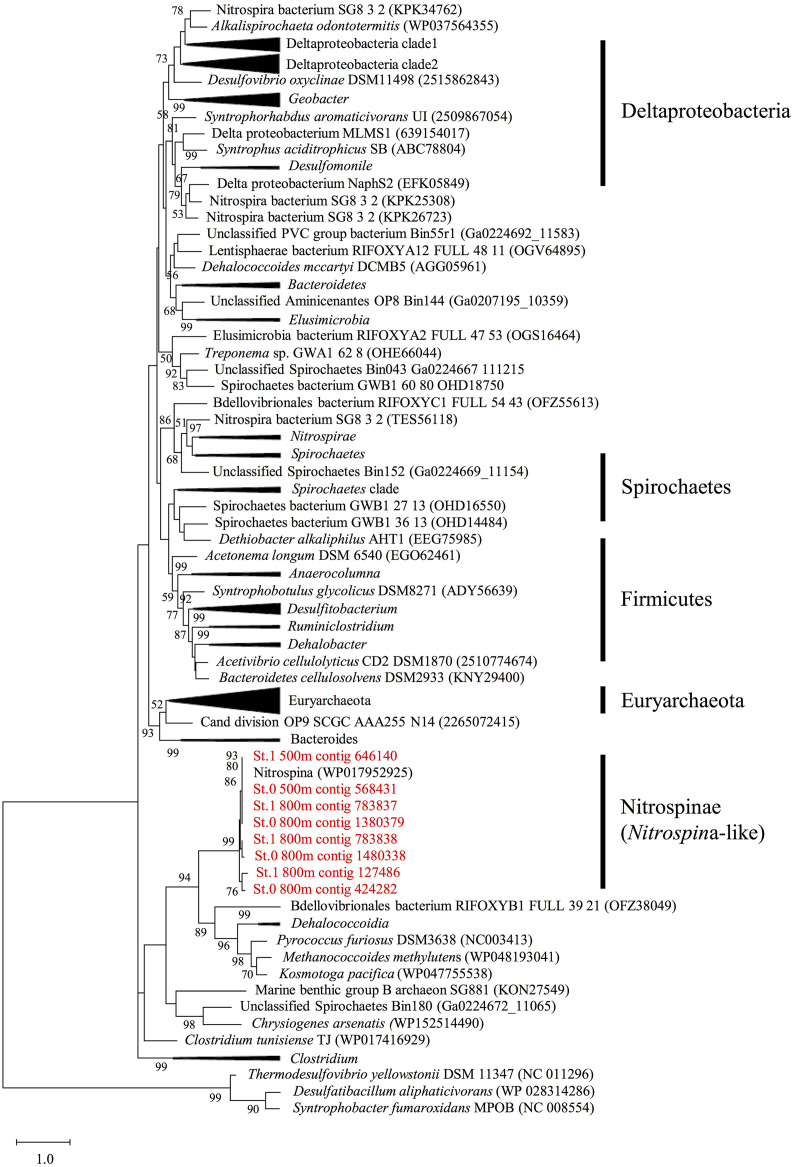
Maximum likelihood phylogenetic tree of the *hgcA* sequences identified in the assembled contigs obtained from mesopelagic layers (depths of 500 and 800 m) at Stations 0 and 1 in the East China Sea. The sequences identified in this study (shown in red) were compared with the *hgcA* homologs described in previous studies (Gilmour et al., 2013, 2018; Gionfriddo et al., 2016; Christensen et al., 2019; Jones et al., 2019). The tree is rooted on *hgcA* paralogs from non-methylators. Bootstrap values > 50 are shown, with consensus based on 100 replicates. The National Center for Biotechnology Information accession numbers or Integrated Microbial Genomes predicted gene numbers are provided.

After assembly using MEGAHIT, we identified eight *hgcA* sequences (four complete and four partial sequences) with lengths greater than 175 amino acids and a cap helix among the assembled contigs detected in the mesopelagic layers (depths of 500 and 800 m) of Sts. 0 and 1 (Supplementary Tables S5, S6). However, none of these sequences were detected in the shallow waters of these stations or throughout the water columns of Sts. 4 and 5. When compared with the *hgcA* gene homologs from experimentally confirmed and predicted methylators (Gilmour et al., 2013; Parks et al., 2013; Gionfriddo et al., 2016; Gilmour et al., 2018; Christensen et al., 2019; Jones et al., 2019), all *hgcA* sequences detected in the present study were found to be closely related to the *Nitrospina* lineages (Figure 5). Moreover, most of the *hgcA* sequences (43 out of 62) detected in the paired-end reads were also closely related to the *Nitrospina* lineages (Supplementary Figure S6). Additionally, *Dehalococcoidia*-like *hgcA* had been identified in the paired-end reads from the metagenomic sequencing data.

Although *hgcB* sequences affiliated with those in *Nitrospina* were also detected in the assembled contigs and paired-end reads collected from the same mesopelagic depths (Supplementary Figures S5–S8), we could not find *hgcB* sequences affiliated with *Dehalococcoidia*.

### Abundance of Functional Modules

**FIGURE 6:**
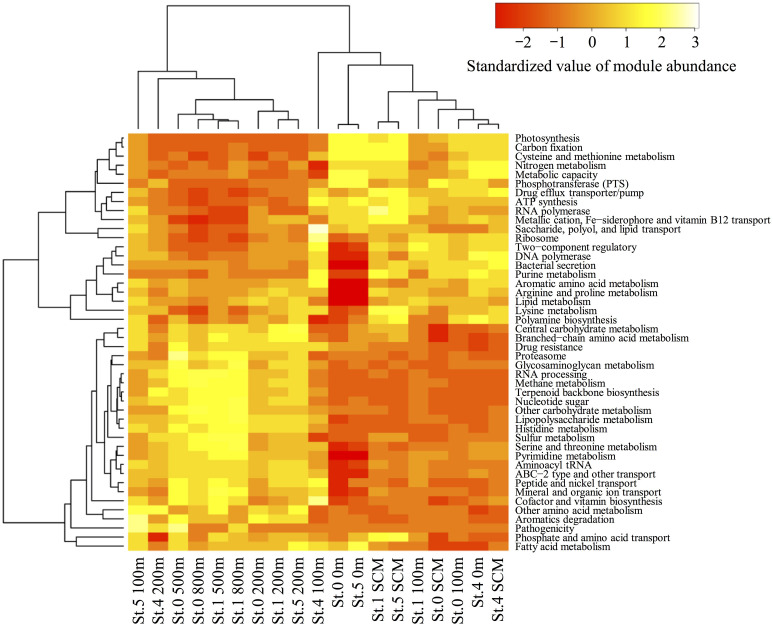
Heatmap of the relative functional module abundances determined using the MAPLE analysis with the paired-end reads from the metagenomic data. The values of module abundance are standardized with average and standard deviations. The module abundance was normalized with reference to ribosomal protein abundance.

Using MAPLE, we identified differences in functional modules between the epipelagic and the mesopelagic layers in the survey area (Figure 6) (the raw data are presented in Supplementary Table S7). We found that the functional modules such as photosynthesis, carbon fixation, and nitrogen metabolism tended to be abundant in the surface layers, whereas the modules associated with sulfate-reducing and methane metabolism (presumably associated with MeHg accumulation) were more prominent in the mesopelagic layers. Notably, we found that the MCR of the functional modules associated with reductive acetyl-CoA pathway involved in Hg methylation (Choi et al., 1994) reached up to 80% at depths of 800 and 500 m in Sts. 0 and 1, respectively (Table 2).

**TABLE 2:** Abundance of Kyoto Encyclopedia of Genes and Genomes orthologous genes involved in the acetyl-CoA pathway (analyzed by the MAPLE software with three million predicted gene sequences from each sample).

Definition	KEGG ortholog	Abundance of KEGG orthologous genes within 3 million predicted gene sequences																		
		St. 0						St. 1					St. 4				St. 5			
		0 m	SCM	100 m	200 m	500 m	800 m	SCM	100 m	200 m	500 m	800 m	0 m	SCM	100 m	200 m	0 m	SCM	100 m	200 m
Anaerobic carbon-monoxide dehydrogenase catalytic subunit (EC:1.2.7.4)	K00198	0	0	0	0	0	2	0	0	0	0	0	0	0	0	0	0	0	0	0
Formate dehydrogenase (NADP+) alpha subunit (EC:1.17.1.10)	K05299	15	19	18	8	72	64	9	27	12	81	83	12	1	9	28	11	13	63	81
Formate-tetrahydrofolate ligase (EC:6.3.4.3)	K01938	1877	1317	133	133	123	19	1359	1412	1221	125	1139	119	1251	1788	15	1857	1412	127	1149
Methylenetetrahydrofolate dehydrogenase (NADP+)/methenyltetrahydrofolate cyclohydrolase (EC:1.5.1.5 3.5.4.9)	K01491	1585	1398	1499	1268	1144	1188	1638	1557	1163	1194	126	1464	1553	1625	126	1678	161	1265	186
Methylenetetrahydrofolate reductase (NADPH) (EC:1.5.1.20)	K00297	1395	11	13	1123	16	149	15	133	125	1145	1199	126	1113	1355	922	1368	1178	981	954
5-Methyltetrahydrofolate corrinoid/iron sulfur protein methyltransferase (EC:2.1.1.258)	K15023	0	0	0	4	6	2	0	0	3	3	1	0	0	1	2	0	0	0	3
Acetyl-CoA synthase (EC:2.3.1.169)	K14138	0	0	0	0	0	0	0	0	0	1	0	0	0	0	0	0	0	0	0
Acetyl-CoA decarbonylase/synthase complex subunit gamma [EC:2.1.1.245]	K00197	0	0	0	0	0	1	0	0	0	1	0	0	0	0	0	0	0	0	0
Acetyl-CoA decarbonylase/synthase complex subunit delta (EC:2.1.1.245)	K00194	0	0	0	0	0	0	0	0	0	0	0	0	0	0	0	0	0	0	0
Glycine hydroxymethyltransferase (EC:2.1.2.1)	K00600	2815	1938	246	1775	1642	1759	2272	1968	1757	1857	1833	211	282	2113	1814	2882	2156	181	162
Module completion ratio (MCR) (%)		50	50	50	60	60	80	50	50	60	80	60	50	50	60	60	50	50	50	60

## Discussion

### MeHg Accumulation by Microorganisms in the Seawater Column

Although we did not sample the bottom water at Sts. 0 and 1, we found that the concentration of MeHg increased with depth in the area that we surveyed in this study. This finding suggests that there might be a pool of MeHg in the mesopelagic layers in this region, which is consistent with the findings of previous studies (Sunderland et al., 2009; Malcolm et al., 2010; Mason et al., 2012; Munson et al., 2015; Kim et al., 2017). In addition, the MeHg/THg ratio changed with depth, with MeHg accounting for a relatively high proportion of THg in the mesopelagic layers at Sts. 0 and 1. In general, MeHg is known to be produced *via* the microbial remineralization of organic matter, associated with sinking particles transported from the surface to the deep ocean layers (Cossa et al., 2009; Sunderland et al., 2009; Heimbürger et al., 2010; Blum et al., 2013; Kim et al., 2017). Also, it has been suggested that the variations in MeHg concentrations in some coastal areas can be affected by riverine inputs and MeHg production within sediments (Hammerschmidt and Fitzgerald, 2008; Lehnherr et al., 2011; Schartup et al., 2014). However, the findings of the present study indicated that the predominant chemical forms of Hg change from surface to deep layers and that MeHg could be accumulated in the mesopelagic layers of the ECS.

In the metagenomic part of our study, we used the relative abundances of *hgcAB* genes, which were calculated by comparing them to the abundances of *recA* genes, to evaluate the distributions of these genes in the seawater column. However, to evaluate the relationships between MeHg and *hgcAB* distributions more precisely, it is better to estimate the absolute abundances of these genes in the metagenomic DNA at each depth.

Our metagenomic analysis revealed that the *hgcA* sequences were detected in the mesopelagic layers, associated with higher MeHg concentrations. Indeed, a significant positive correlation between *hgcA* sequence abundance and MeHg concentrations (Rho = 0.85, *P* < 0.001, *n* = 11) was observed at the deep-water stations (Sts. 0 and 1) (Table 3 and Supplementary Figure S9). In addition, the *hgcB* gene sequences were more abundant in the layer deeper than 200 m and weakly correlated with MeHg/THg ratio using data from all stations (Rho = 0.46, *P* < 0.05, *n* = 19). However, there was no significant correlation between *hgcB* abundance and MeHg concentration. These data indicated that the abundance of the *hgcA* gene could be useful for evaluating the microbial MeHg accumulation in the pelagic seawater column. In a few previous studies, a significant positive relationship between *hgcA* abundance and MeHg concentration has been reported in paddy soils (Liu et al., 2014) and dam sediments (Du et al., 2017) in China. These data thus verify the utility of *hgcA* quantification *via* metagenomic analysis for evaluating the potential of microbial MeHg accumulation in natural environments, including seawater columns.

**TABLE 3:** Spearman’s rank correlation analysis of mercury species and abundance of *hgcAB*-like sequences at all stations and at deep stations (Sts. 0 and 1).

Factors	*Rho* value			
	All stations		Deep stations (Sts. 0 and 1)	
	*hgcA* (*n* = 19)	*hgcB* (*n* = 19)	*hgcA* (*n* = 11)	*hgcB* (*n* = 11)
THg	0.55 (*P* < 0.05)	0.46 (*P* < 0.05)	0.83 (*P* < 0.005)	0.35 (*P* > 0.05)
MeHg	0.73 (*P* < 0.001)	0.45 (*P* > 0.05)	0.85 (*P* < 0.001)	0.35 (*P* > 0.05)
MeHg/THg	0.73 (*P* < 0.001)	0.46 (*P* < 0.05)	0.85 (*P* < 0.001)	0.38 (*P* > 0.05)

It is well known that both *hgcAB* genes are indispensable to microbial mercury methylation (Parks et al., 2013). However, *hgcB* is more abundant in the seawater column than *hgcA*. One possible reason is the distinct detection efficiency between these two genes, which is attributed to the length of the reference sequences in the HMMER analysis. When the target sequences were short, their detection efficiency in the metagenomic reads potentially tended to increase. In our HMM references, the *hgcA* sequences (average 349 AAs) were longer than the *hgcB* sequences (average 99 AAs) (Supplementary Tables S2, S3). This bias could lead to the distinct correlation efficiency between these two genes. Thus, more abundant and longer query sequences would be essential for *hgcAB* distribution and diversity analyses in the oceanic environments.

In the bottom layer of St. 4, a relatively high MeHg concentration was observed. Our data on seawater characteristics (increases in nutrient concentrations) and vertical THg and MeHg profiles (Figures 2, 3) indicate that the bottom water at St. 4 might be affected by marine sediments on the continental shelf or vertical diffusion of deep waters. Some previous studies have reported relatively high THg and MeHg concentrations in the bottom waters around the continental shelves, and these Hg species could be derived from sediments (e.g., 6.6–12.0 pmol m^–2^ day^–1^ in southern New England and 0–2.2 pmol m^–2^ day^–1^ in the mid-Atlantic) (Hammerschmidt and Fitzgerald, 2006; Hollweg et al., 2010). Another study has shown that the upward diffusion from the mesopelagic layers is an important MeHg source in the subsurface waters (1.8–12.0 nmol m^–2^ year^–1^ in the western Pacific Ocean) (Kim et al., 2017). However, our metagenomic data showed no substantial increase in the abundance of *hgcA* in this station, suggesting that the diffusion from deep water in Sts. 0 and 1 could be relatively low. Although there are no physicochemical data on sediment diffusion, it is speculated that the bottom water at St. 4 can be affected by the flux of MeHg from the sediments in the continental shelf.

### *Nitrospina* as a Potential Hg Methylator in the ECS

Most of the *hgcAB* sequences from the paired-end reads and the assembled contigs detected in the mesopelagic layers of the ECS were closely related to those of *Nitrospina* (Figure 5 and Supplementary Figures S5–S8), which have also been detected in Antarctic sea ice, characterized by high concentrations of MeHg (Gionfriddo et al., 2016), the equatorial North Pacific (Bowman et al., 2020), and the global ocean (Villar et al., 2020). In addition, our 16S rRNA deep-sequencing analysis revealed a relatively high abundance of Nitrospinae sequences in the mesopelagic layers of Sts. 0 and 1, where high concentrations of MeHg were recorded. These data indicate that *Nitrospina* may be one of the key phylogenetic lineages associated with the microbial accumulation of MeHg in the ECS water column. *Nitrospina* are bacteria assumed to be major nitrite oxidizers (aerobically and anaerobically) in oceanic mesopelagic layers (Pachiadaki et al., 2017; Sun et al., 2019). Previous studies have tended to indicate that anaerobic prokaryotes among the SRB, IRB, and methanogens are more likely to be the major Hg methylators in aquatic ecosystems (e.g., Bravo et al., 2018). A meta-omics study using the Tara Oceans database revealed that the *Nitrospina*-like *hgcA* gene is widely distributed in the global ocean (Villar et al., 2020). To date, there has been no confirmation, either in the laboratory or in the field, whether species of *Nitrospina* are associated with the aerobic methylation of Hg, and *Nitrospina* carrying *hgcAB* genes have never been cultured. Thus, in future studies, combined culture-dependent and -independent (such as molecular techniques) analyses will be essential for estimating the contribution of *Nitrospina* to ocean MeHg accumulation.

The phylogenetic analysis using paired-end reads revealed that *Dehalococcoidia* (affiliated with Chloroflexi)-like *hgcA* were detected from the mesopelagic layers at a depth of 800 m in St. 0 and at depths of 500 and 800 m in St. 1 (Supplementary Figures S6, S7). This lineage obtains energy *via* reductive dehalogenation (e.g., Wagner et al., 2012), and some species within this lineage harbor *hgcAB* genes (Gilmour et al., 2013). In a previous metagenomic study, *Dehalococcoidia*-like *hgcA* was detected in the sediments and waters of aquatic environments (Podar et al., 2015). However, any *hgcB* sequences affiliated with *Dehalococcoidia* were not observed in the paired-end reads and the assembled metagenomic sequences from the mesopelagic layers in this study (Supplementary Figures S5–S8). *Vice versa*, several sequences of *Syntrophus*-like *hgcB* were detected in the paired-end reads from the metagenomic data, but those of *hgcA* were not. These data suggest that the contribution of *Dehalococcoidia* and *Syntrophus* lineages toward MeHg accumulation may be smaller than that of the *Nitrospina* lineage in the mesopelagic layers of the ESC.

The 16S rRNA deep-sequencing analysis conducted in the present study also revealed that Deltaproteobacteria are abundant in the mesopelagic layers (Figure 4, Supplementary Table S4, and Supplementary Figure S2). Specifically, the Deltaproteobacteria *Desulfobulbaceae* and *Desulfuromonadaceae* are among the major SRBs in coastal sediments (Gittel et al., 2008; Leloup et al., 2009; Ihara et al., 2019), and some species from these lineages are Hg methylators or *hgcAB* carriers (Gilmour et al., 2013; Christensen et al., 2019). However, the contributions of these lineages could be smaller than that of the Nitrospinae lineage (Supplementary Figures S3, S4), given that no deltaproteobacterial *hgcA* sequences were detected in these layers. These data are consistent with the hypothesis that the deltaproteobacterial methylators affiliated with some members of SRB have only a minor contribution to MeHg accumulation in the seawater columns of the ECS.

The increases of *hgcB* abundance were observed at a depth of 200 m, in a layer with high salinity and nitrite concentrations (Figures 2B,E, 3F). However, the *hgcA* sequences were not detected in these layers, potentially due to the low detection efficiency described above. The phylogenetic analyses for *hgcB* in the metagenomes obtained from Sts. 0 and 1 showed that the *hgcB* sequences of *Nitrospina* (including nitrite oxidizing lineages) were identified at depths of 200 m in both stations (Supplementary Figure S8). Furthermore, several unknown *hgcAB* sequences were detected throughout the seawater column. Thus, further analyses using HMM references to obtain more diverse and high-quality *hgcAB* sequences could lead to a better understanding of the distribution and the diversity of *hgcAB* genes in the ocean.

Metagenomic analysis is one of the powerful tools to survey the distribution of specific functional genes such as *hgcAB* in marine environments. However, a combination analysis with the metatranscriptome might be a better approach to assess the relationship between functional genes and trace metal dynamics. The transcriptome analysis can be used to evaluate the expression of functional genes to provide more quantitative data. These combination analyses of the Hg methylation genes revealed that the *Nitrospina*-like *hgcA* is actively expressed in the oxic open ocean (Villar et al., 2020). Thus, further combination analyses with metagenome and metatranscriptome would lead to a better understanding of the *hgcAB* genes and mercury and MeHg distribution in the ocean.

### Metabolic Pathways Associated With MeHg Accumulation in Seawater

A metabolic pathway analysis using MAPLE revealed significant differences in the abundance of functional modules between surface and deep layers in the seawater column (Figure 6). In particular, the modules of sulfur and methane metabolism associated with SRB and methanogens were relatively more abundant in the deeper layers (notably at depths of 500 and 800 m in Sts. 0 and 1) than in the surface layers. The results of the 16S rRNA deep-sequencing analysis revealed that sequences affiliated with *Syntrophobacteraceae* (members of SRB) and *Thermoplasmata* (members of methanogens) are abundant in mesopelagic layers (Supplementary Figure S4). In contrast, these trends were not observed in *Desulfobulbaceae*, *Desulfuromonadaceae*, and *Bdellovibrionaceae* (SRB, including some Hg methylators or *hgcAB* carriers). The partial 16S rRNA gene analysis cannot completely detect the Hg methylators because *hgcAB* presence/absence varies within a species. However, we were unable to detect any *hgcA* sequences associated with the SRB and the methanogens in the mesopelagic layers. These data suggested that there might not be a tight linkage between MeHg accumulation and the sulfur or methane metabolism of SRB and methanogens in the ECS.

Our MAPLE analysis also revealed that the modules associated with the reductive acetyl-CoA pathway were not completely filled with genes at all stations and depths (Table 2). In general, the insufficient coverage of query sequences might have an effect on the completion ratio of genes and modules. The results of the rarefaction curve in the MAPLE analysis showed that the gene and module detection was close to saturation (Supplementary Figure S11). Thus, our results suggested that the activity of this pathway in anaerobic microbes such as SRBs and methanogens might not be substantial in the ECS seawater columns. The findings of some previous studies have, nevertheless, indicated that this pathway could be associated with Hg methylation and HgcAB proteins (Choi et al., 1994; Parks et al., 2013). Taken together with our MAPLE results and *hgcAB* distribution, anaerobic microbes such as SRBs and methanogens might constitute a minor fraction of MeHg accumulation in the ECS water column.

To search specific functional genes in the metagenomic data, the abundance and the coverage of query sequences are critical factors in evaluating the presence or the absence of genes. In this study, we could not detect substantial numbers of *hgcAB* sequences in the metagenomic reads for the diversity analysis of these genes (Supplementary Table S5). The results of the rarefaction curves for *hgcAB* OTUs (>90% similarity) in the paired-end reads showed that the number of OTUs did not plateau in our metagenomic data (Supplementary Figure S12). Therefore, further analysis with adequate query sequences is needed to evaluate the Hg methylation gene abundance and diversity among the marine environments.

### Other Potential Processes of MeHg Accumulation in the ECS

As with the *in situ* microbial MeHg production and the sediment source, physical factors, such as the diffusion flux, can be critical for the distribution of MeHg in the ocean. A recent extensive survey of THg and MeHg in the Western Pacific Ocean revealed that the diffusion flux and the distribution of MeHg could be affected by ocean circulation (Kim et al., 2017). In the ECS, the Kuroshio Current is known to effectively transport organic and inorganic materials (e.g., Guo et al., 2012). In previous studies conducted in the ECS, the DO concentrations at bottom waters (1000–1500 m in depth) were 2.6–2.9 mg L^–1^ (80–100 μmol kg^–1^) (Wong et al., 1991; Guo et al., 2012), which were similar to those of the mesopelagic layers in this study (Figure 2C). These data suggest that the high concentrations of MeHg in the mesopelagic layers in Sts. 0 and 1 were unlikely due to the diffusion of bottom water.

MeHg degradation is also an important factor in the regulation of MeHg in the seawater column. It is well known that photochemical and microbial processes contribute to MeHg demethylation in aquatic environments (Fitzgerald et al., 2007; Mason et al., 2012). In this study, MeHg depletion was observed in the surface layers, in which the *hgcB* sequences were detected. These data suggest that there was a potential for photochemical degradation of MeHg derived from microorganisms. In terms of the microbial processes involved, previous studies have reported that the mercury resistance (*mer*) operon is involved in the demethylation of MeHg (Barkay et al., 2003; Brown et al., 2003), and a variety of Bacteria and Archaea possess it (Boyd and Barkay, 2012). Thus, further combination analyses on photochemical and microbial MeHg degradation would provide a better understanding on the distributions of Hg and MeHg in the ECS.

## Conclusion

In the present study, we used metagenomic approaches to examine the distribution patterns of *hgcAB* sequences in the water column of the ECS. We found that *hgcA* sequences were abundant in the mesopelagic layers and that the relative abundance of these sequences was positively correlated with the concentrations of dissolved MeHg. Notably, we detected a significant positive correlation between *hgcA* abundance relative to *recA* and MeHg concentrations, thereby indicating that the quantification of *hgcA* sequences in the water column could be valuable for evaluating the microbial accumulation of MeHg in marine environments, except for the continental shelf area influenced by sediment Hg. The *hgcA* sequences detected in the mesopelagic layers were phylogenetically close to those of bacteria within the *Nitrospina* lineage, which are known as aerobically and anaerobically nitrite-oxidizing marine bacteria. In addition, we also detected sequences of *Nitrospina*-like *hgcB* in the same water layers. Metabolic pathway analysis based on MAPLE revealed that modules related to sulfur and methane metabolism were prominent in the mesopelagic layers. However, we were unable to detect any *hgcA* sequences affiliated with SRB or methanogens. Collectively, our results indicate that species of aerophilic marine bacteria within the *Nitrospina* lineage carrying *hgcAB* genes could be important mediators of MeHg accumulation in the mesopelagic layers of the ECS. More extensive surveys of Hg methylation and MeHg demethylation genes based on metagenomics and metatranscriptomics approaches will enable us to have a better understanding of the contribution of marine microorganisms to MeHg accumulation in marine ecosystems.

## Data Availability Statement

The datasets generated for this study can be found in the DRA009218 and DRA008415.

## Author Contributions

YT, KM, and AT contributed to conception and design of the study. YT organized the main metagenome database. KM and AT performed the mercury and methylmercury analyses. YT wrote the first draft of the manuscript. All authors contributed to manuscript revision and read and approved the submitted version. The corresponding author takes primary responsibility for communication with the journal and editorial office during the submission process, throughout peer review, and during publication. The corresponding author is also responsible for ensuring that the submission adheres to all journal requirements including, but not exclusive to, details of authorship, study ethics and ethics approval, clinical trial registration documents, and conflict of interest declaration. The corresponding author should also be available post-publication to respond to any queries or critiques.

## Conflict of Interest

The authors declare that the research was conducted in the absence of any commercial or financial relationships that could be construed as a potential conflict of interest.

## Footnotes

1 http://qiime.org/index.html

2 http://hannonlab.cshl.edu/fastx_toolkit/

3 https://maple.jamstec.go.jp/maple/maple-2.3.1
