# Supplementary material for: Nitrospina-Like Bacteria Are Potential Mercury Methylators in the Mesopelagic Zone in the East China Sea
Source: Front Microbiol. 2020 Jul 3;11:1369. doi: 10.3389/fmicb.2020.01369 (PMC7347909; doi:10.3389/fmicb.2020.01369)
Supplement: Supplementary file 1 [file Presentation_1.PPT]

## Slide 1
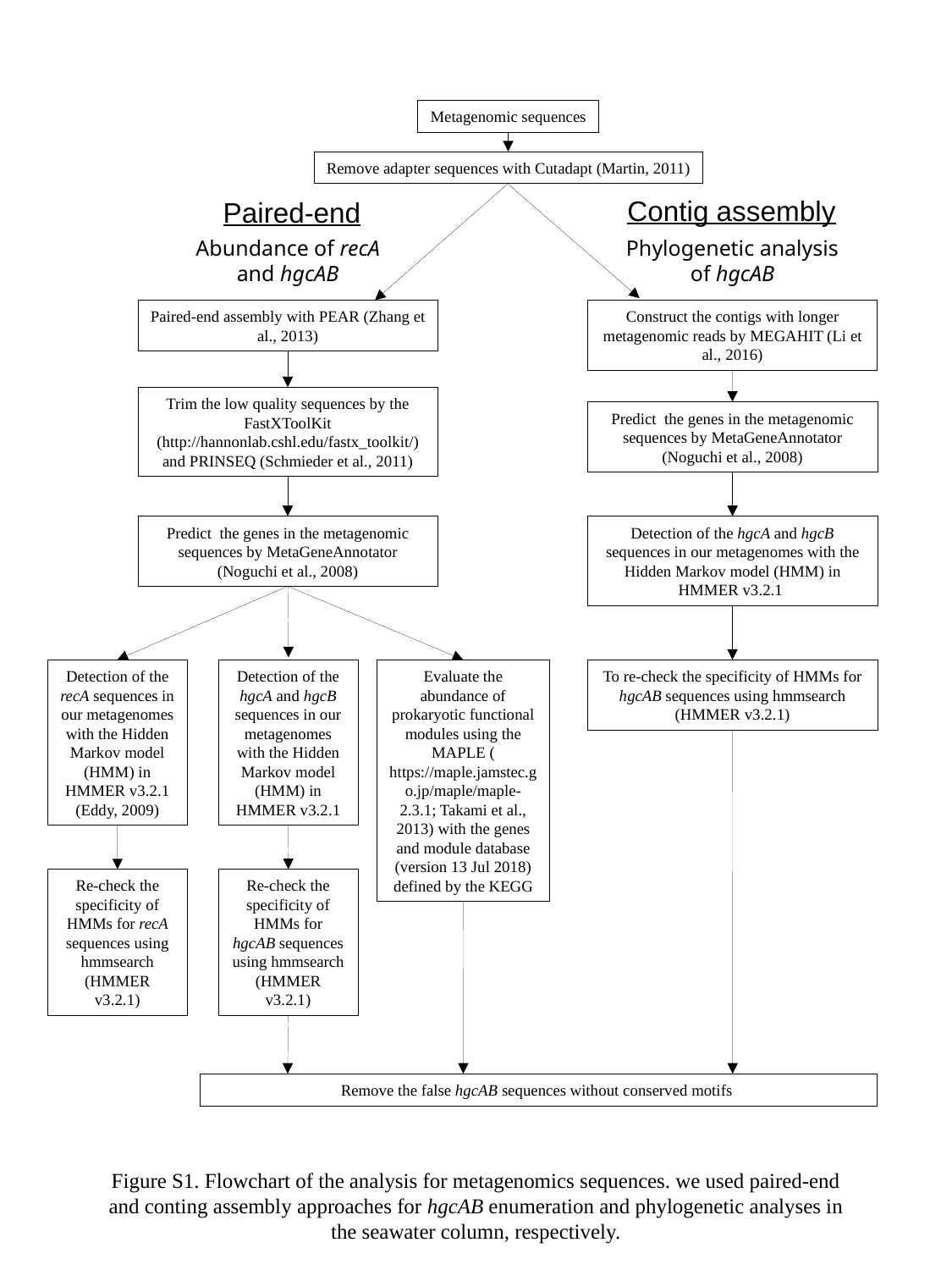

Metagenomic sequences
Remove adapter sequences with Cutadapt (Martin, 2011)
Contig assembly
Paired-end
Abundance of recA and hgcAB
Phylogenetic analysis of hgcAB
Paired-end assembly with PEAR (Zhang et al., 2013)
Construct the contigs with longer metagenomic reads by MEGAHIT (Li et al., 2016)
Trim the low quality sequences by the FastXToolKit (http://hannonlab.cshl.edu/fastx_toolkit/) and PRINSEQ (Schmieder et al., 2011)
Predict the genes in the metagenomic sequences by MetaGeneAnnotator (Noguchi et al., 2008)
Predict the genes in the metagenomic sequences by MetaGeneAnnotator (Noguchi et al., 2008)
Detection of the hgcA and hgcB sequences in our metagenomes with the Hidden Markov model (HMM) in HMMER v3.2.1
Detection of the recA sequences in our metagenomes with the Hidden Markov model (HMM) in HMMER v3.2.1 (Eddy, 2009)
Detection of the hgcA and hgcB sequences in our metagenomes with the Hidden Markov model (HMM) in HMMER v3.2.1
Evaluate the abundance of prokaryotic functional modules using the MAPLE ( https://maple.jamstec.go.jp/maple/maple-2.3.1; Takami et al., 2013) with the genes and module database (version 13 Jul 2018) defined by the KEGG
To re-check the specificity of HMMs for hgcAB sequences using hmmsearch (HMMER v3.2.1)
Re-check the specificity of HMMs for recA sequences using hmmsearch (HMMER v3.2.1)
Re-check the specificity of HMMs for hgcAB sequences using hmmsearch (HMMER v3.2.1)
Remove the false hgcAB sequences without conserved motifs
Figure S1. Flowchart of the analysis for metagenomics sequences. we used paired-end and conting assembly approaches for hgcAB enumeration and phylogenetic analyses in the seawater column, respectively.

## Slide 2
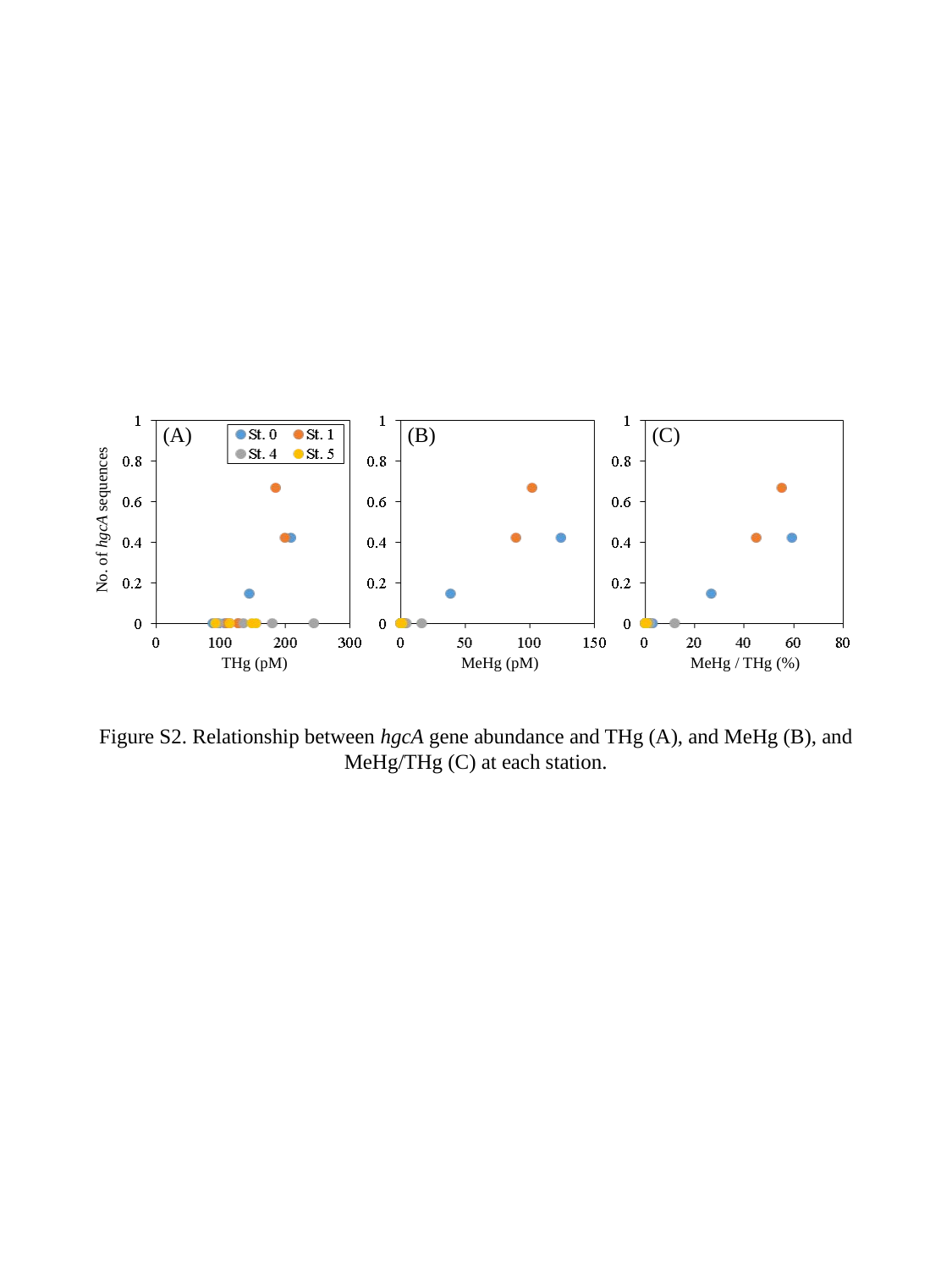

(A)
(B)
(C)
No. of hgcA sequences
THg (pM)
MeHg (pM)
MeHg / THg (%)
Figure S2. Relationship between hgcA gene abundance and THg (A), and MeHg (B), and MeHg/THg (C) at each station.

## Slide 3
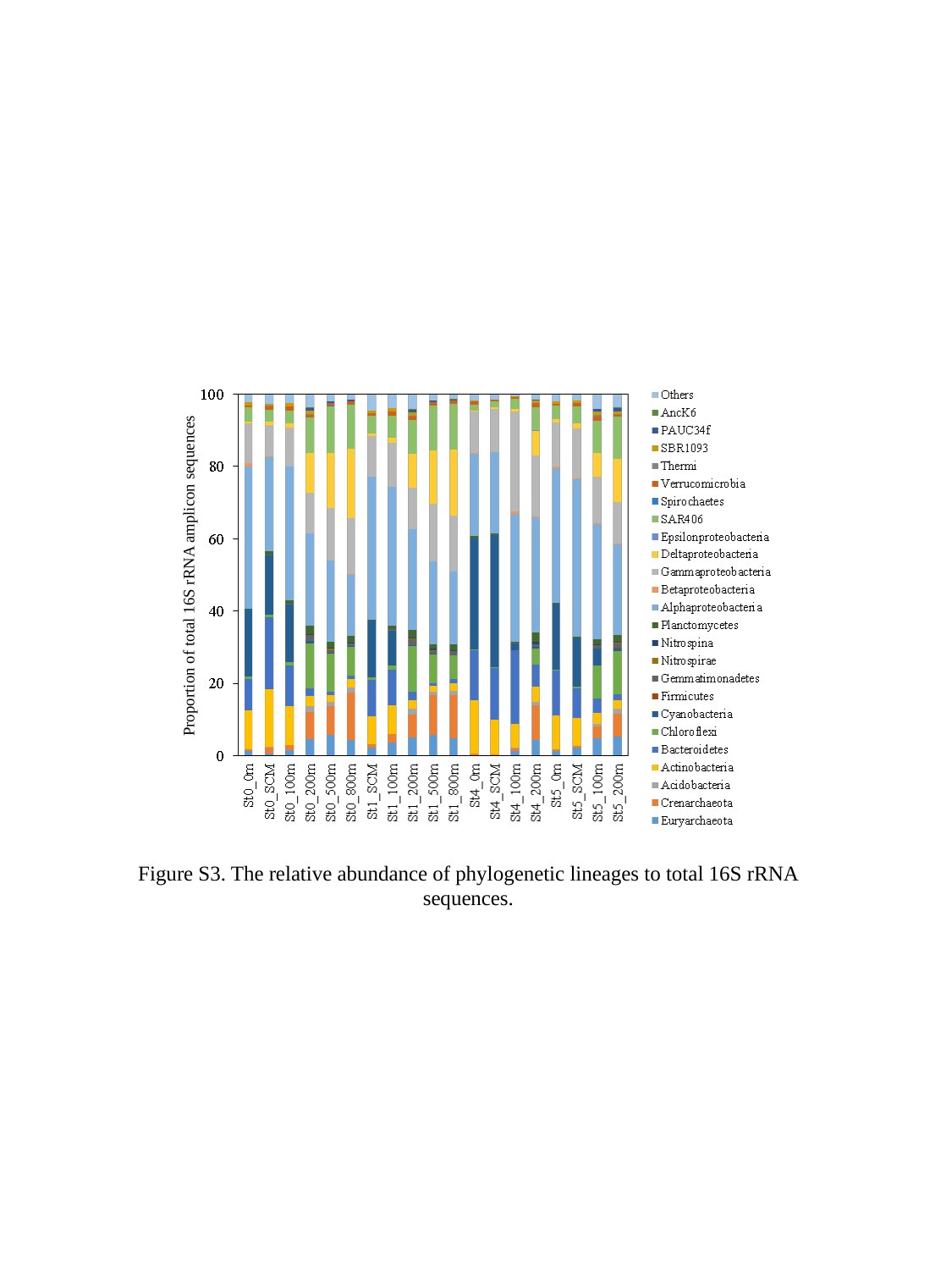

Proportion of total 16S rRNA amplicon sequences
Figure S3. The relative abundance of phylogenetic lineages to total 16S rRNA sequences.

## Slide 4
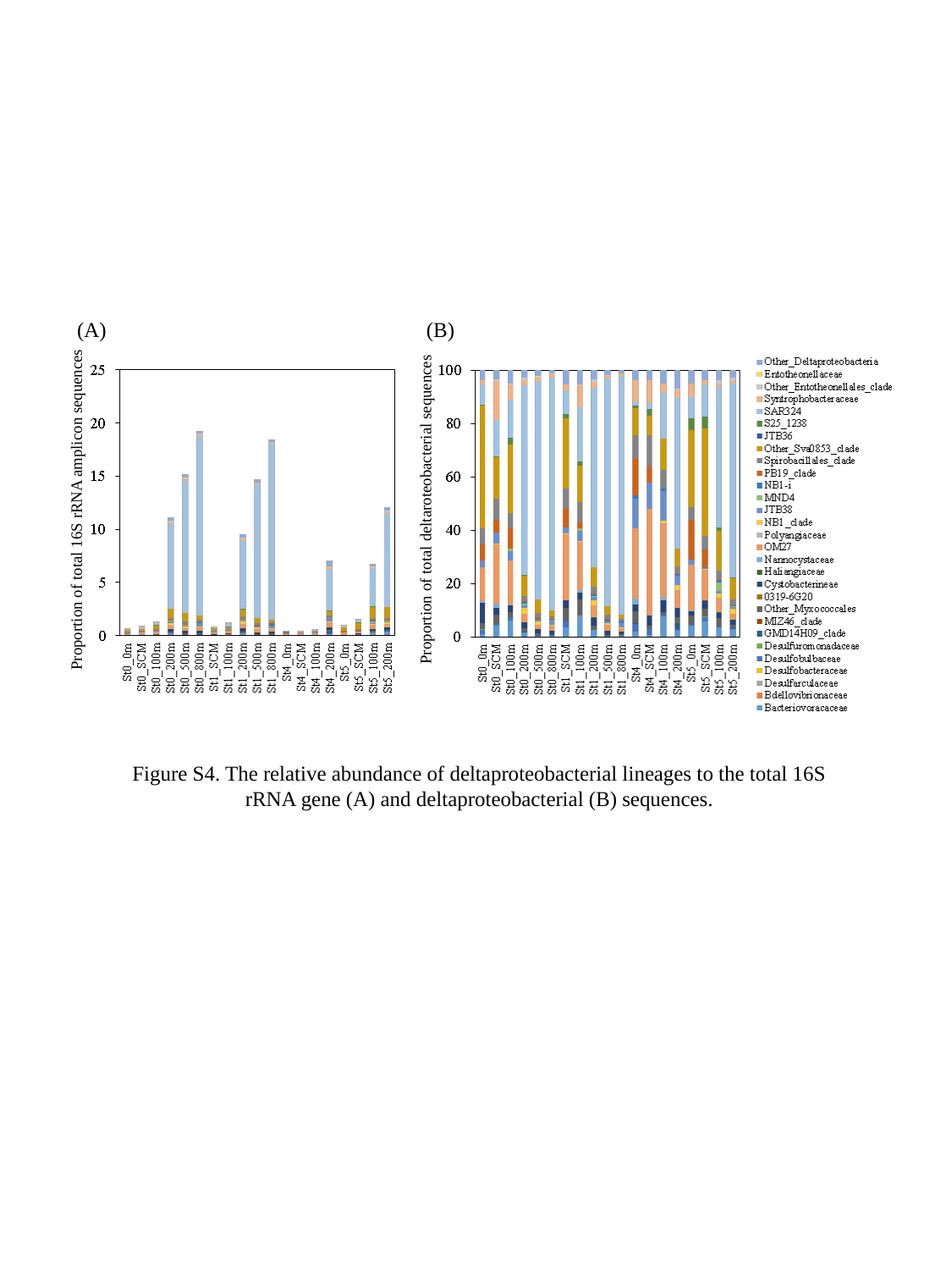

(A)
(B)
Proportion of total deltaroteobacterial sequences
Proportion of total 16S rRNA amplicon sequences
Figure S4. The relative abundance of deltaproteobacterial lineages to the total 16S rRNA gene (A) and deltaproteobacterial (B) sequences.

## Slide 5
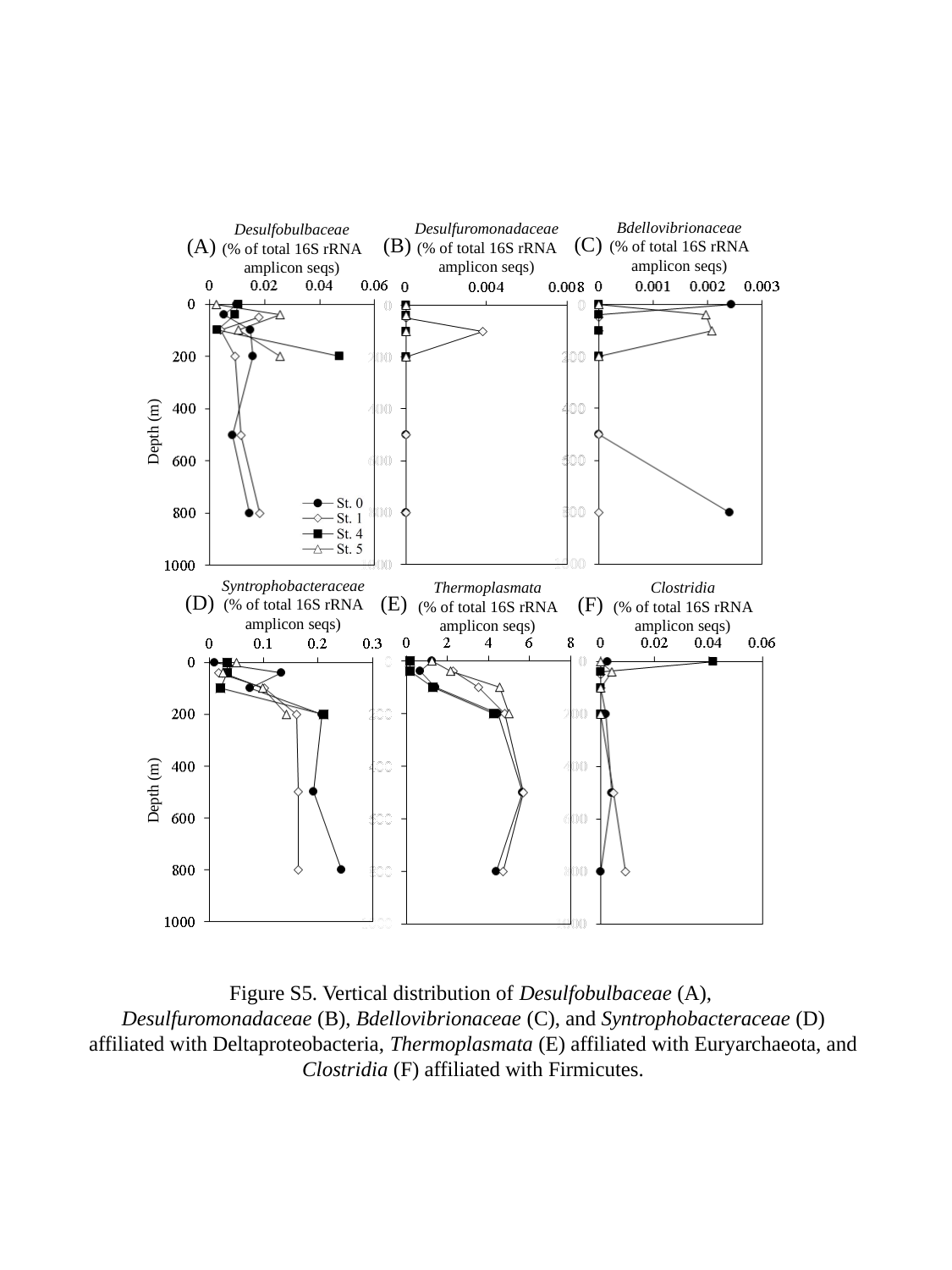

Bdellovibrionaceae
(% of total 16S rRNA amplicon seqs)
Desulfuromonadaceae
(% of total 16S rRNA amplicon seqs)
Desulfobulbaceae
(% of total 16S rRNA amplicon seqs)
(C)
(B)
(A)
Depth (m)
Syntrophobacteraceae
(% of total 16S rRNA amplicon seqs)
Thermoplasmata
(% of total 16S rRNA amplicon seqs)
Clostridia
(% of total 16S rRNA amplicon seqs)
(D)
(E)
(F)
Depth (m)
Figure S5. Vertical distribution of Desulfobulbaceae (A),
Desulfuromonadaceae (B), Bdellovibrionaceae (C), and Syntrophobacteraceae (D) affiliated with Deltaproteobacteria, Thermoplasmata (E) affiliated with Euryarchaeota, and Clostridia (F) affiliated with Firmicutes.

## Slide 6
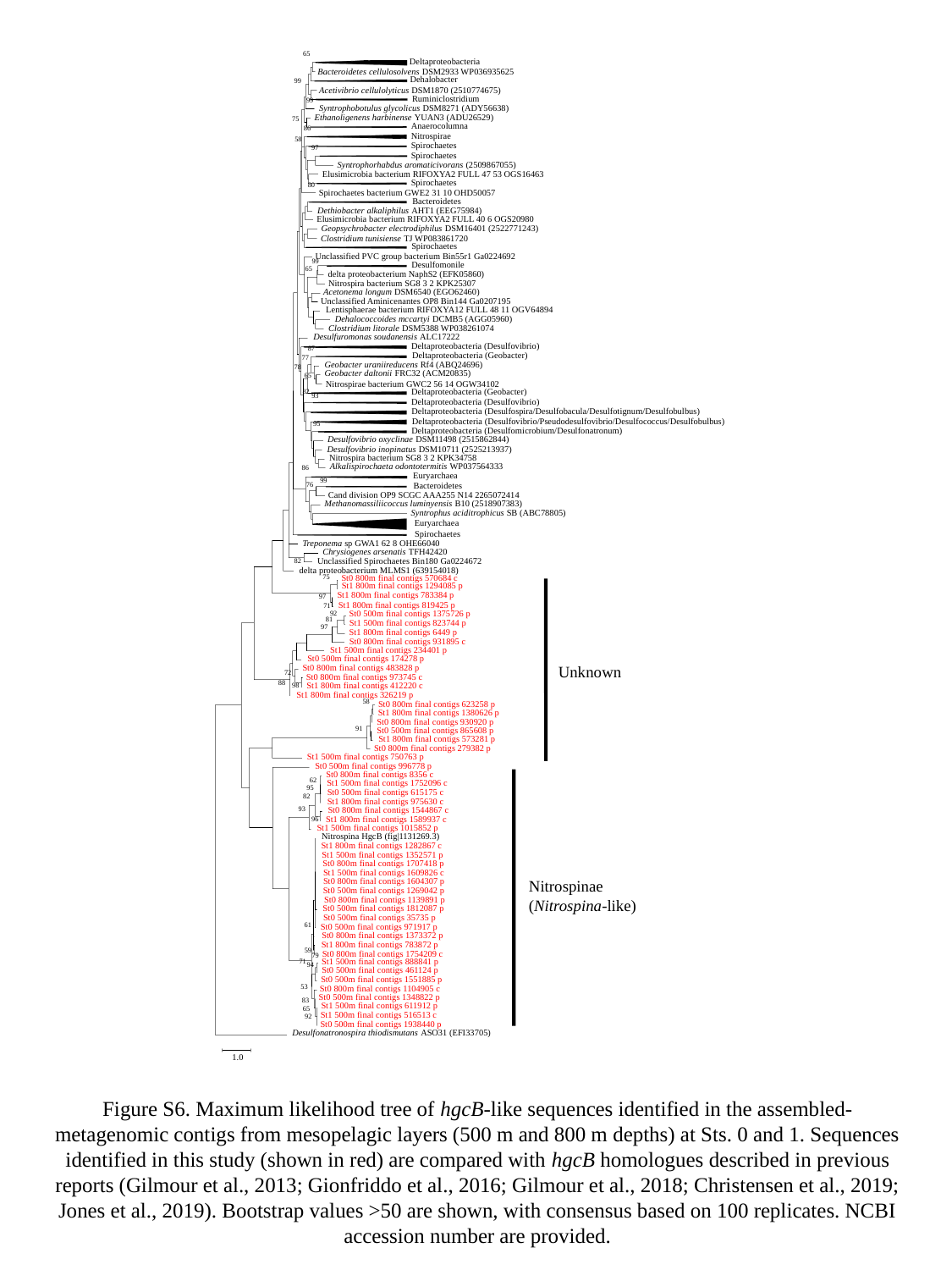

65
Deltaproteobacteria
Bacteroidetes cellulosolvens DSM2933 WP036935625
Dehalobacter
99
Acetivibrio cellulolyticus DSM1870 (2510774675)
Ruminiclostridium
99
Syntrophobotulus glycolicus DSM8271 (ADY56638)
Ethanoligenens harbinense YUAN3 (ADU26529)
75
Anaerocolumna
86
Nitrospirae
58
Spirochaetes
97
Spirochaetes
Syntrophorhabdus aromaticivorans (2509867055)
Elusimicrobia bacterium RIFOXYA2 FULL 47 53 OGS16463
Spirochaetes
80
Spirochaetes bacterium GWE2 31 10 OHD50057
Bacteroidetes
Dethiobacter alkaliphilus AHT1 (EEG75984)
Elusimicrobia bacterium RIFOXYA2 FULL 40 6 OGS20980
Geopsychrobacter electrodiphilus DSM16401 (2522771243)
Clostridium tunisiense TJ WP083861720
Spirochaetes
Unclassified PVC group bacterium Bin55r1 Ga0224692
99
Desulfomonile
65
delta proteobacterium NaphS2 (EFK05860)
Nitrospira bacterium SG8 3 2 KPK25307
Acetonema longum DSM6540 (EGO62460)
Unclassified Aminicenantes OP8 Bin144 Ga0207195
Lentisphaerae bacterium RIFOXYA12 FULL 48 11 OGV64894
Dehalococcoides mccartyi DCMB5 (AGG05960)
Clostridium litorale DSM5388 WP038261074
Desulfuromonas soudanensis ALC17222
Deltaproteobacteria (Desulfovibrio)
87
Deltaproteobacteria (Geobacter)
77
Geobacter uraniireducens Rf4 (ABQ24696)
78
Geobacter daltonii FRC32 (ACM20835)
65
Nitrospirae bacterium GWC2 56 14 OGW34102
Deltaproteobacteria (Geobacter)
82
93
Deltaproteobacteria (Desulfovibrio)
Deltaproteobacteria (Desulfospira/Desulfobacula/Desulfotignum/Desulfobulbus)
Deltaproteobacteria (Desulfovibrio/Pseudodesulfovibrio/Desulfococcus/Desulfobulbus)
95
Deltaproteobacteria (Desulfomicrobium/Desulfonatronum)
Desulfovibrio oxyclinae DSM11498 (2515862844)
Desulfovibrio inopinatus DSM10711 (2525213937)
Nitrospira bacterium SG8 3 2 KPK34758
Alkalispirochaeta odontotermitis WP037564333
86
Euryarchaea
99
Bacteroidetes
76
Cand division OP9 SCGC AAA255 N14 2265072414
Methanomassiliicoccus luminyensis B10 (2518907383)
Syntrophus aciditrophicus SB (ABC78805)
Euryarchaea
Spirochaetes
Treponema sp GWA1 62 8 OHE66040
Chrysiogenes arsenatis TFH42420
Unclassified Spirochaetes Bin180 Ga0224672
82
delta proteobacterium MLMS1 (639154018)
St0 800m final contigs 570684 c
75
St1 800m final contigs 1294085 p
St1 800m final contigs 783384 p
97
St1 800m final contigs 819425 p
71
St0 500m final contigs 1375726 p
92
81
St1 500m final contigs 823744 p
97
St1 800m final contigs 6449 p
St0 800m final contigs 931895 c
St1 500m final contigs 234401 p
St0 500m final contigs 174278 p
Unknown
St0 800m final contigs 483828 p
72
St0 800m final contigs 973745 c
88
St1 800m final contigs 412220 c
98
St1 800m final contigs 326219 p
58
St0 800m final contigs 623258 p
St1 800m final contigs 1380626 p
St0 800m final contigs 930920 p
St0 500m final contigs 865608 p
91
St1 800m final contigs 573281 p
St0 800m final contigs 279382 p
St1 500m final contigs 750763 p
St0 500m final contigs 996778 p
St0 800m final contigs 8356 c
62
St1 500m final contigs 1752096 c
95
St0 500m final contigs 615175 c
82
St1 800m final contigs 975630 c
St0 800m final contigs 1544867 c
93
St1 800m final contigs 1589937 c
96
St1 500m final contigs 1015852 p
Nitrospina HgcB (fig|1131269.3)
St1 800m final contigs 1282867 c
St1 500m final contigs 1352571 p
St0 800m final contigs 1707418 p
St1 500m final contigs 1609826 c
Nitrospinae
(Nitrospina-like)
St0 800m final contigs 1604307 p
St0 500m final contigs 1269042 p
St0 800m final contigs 1139891 p
St0 500m final contigs 1812087 p
St0 500m final contigs 35735 p
St0 500m final contigs 971917 p
61
St0 800m final contigs 1373372 p
St1 800m final contigs 783872 p
59
St0 800m final contigs 1754209 c
79
St1 500m final contigs 888841 p
71
94
St0 500m final contigs 461124 p
St0 500m final contigs 1551885 p
St0 800m final contigs 1104905 c
53
St0 500m final contigs 1348822 p
83
St1 500m final contigs 611912 p
65
St1 500m final contigs 516513 c
92
St0 500m final contigs 1938440 p
Desulfonatronospira thiodismutans ASO31 (EFI33705)
1.0
Figure S6. Maximum likelihood tree of hgcB-like sequences identified in the assembled-metagenomic contigs from mesopelagic layers (500 m and 800 m depths) at Sts. 0 and 1. Sequences identified in this study (shown in red) are compared with hgcB homologues described in previous reports (Gilmour et al., 2013; Gionfriddo et al., 2016; Gilmour et al., 2018; Christensen et al., 2019; Jones et al., 2019). Bootstrap values >50 are shown, with consensus based on 100 replicates. NCBI accession number are provided.

## Slide 7
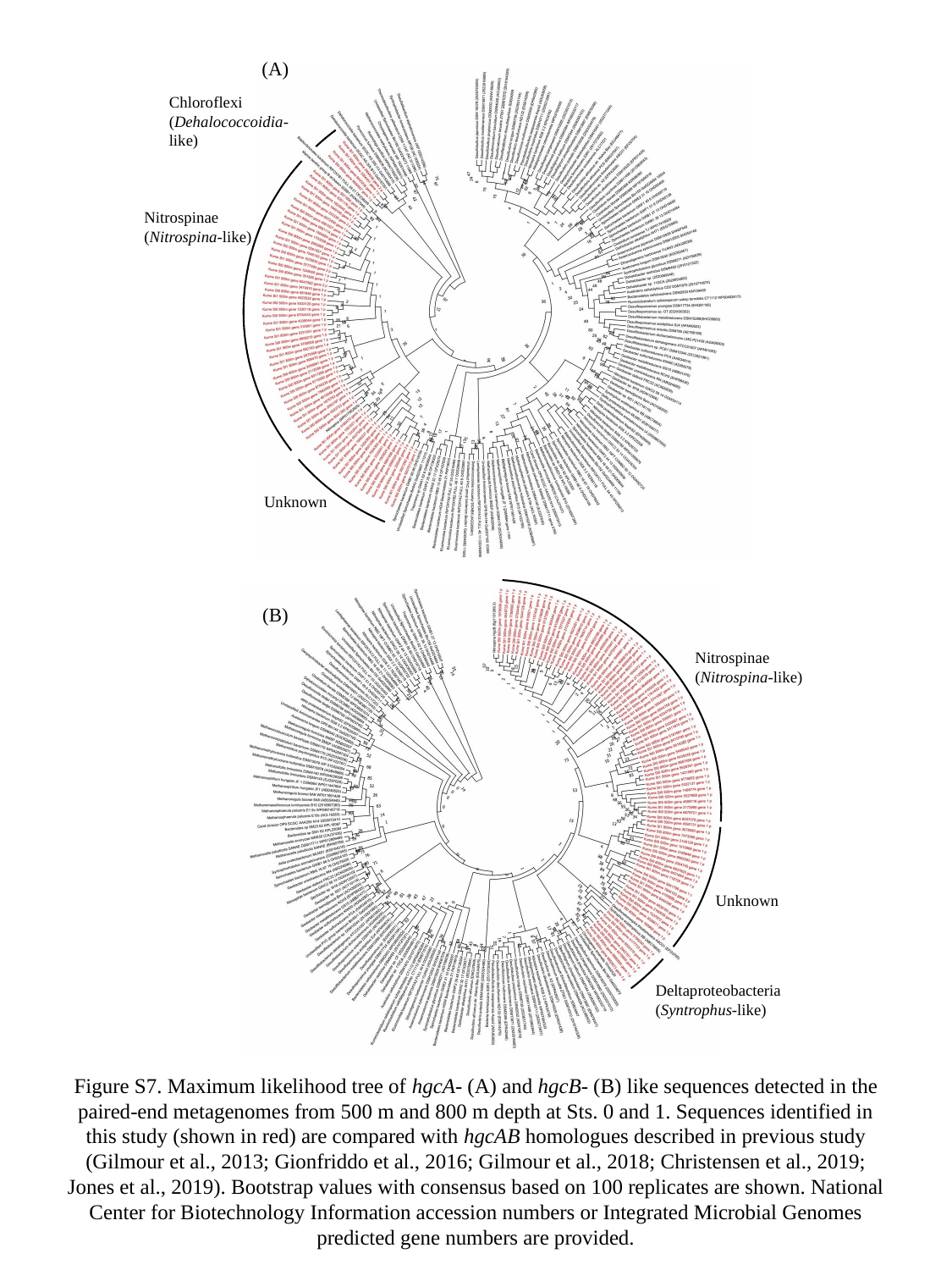

(A)
Chloroflexi
(Dehalococcoidia-like)
Nitrospinae
(Nitrospina-like)
Unknown
(B)
Nitrospinae
(Nitrospina-like)
Unknown
Deltaproteobacteria
(Syntrophus-like)
Figure S7. Maximum likelihood tree of hgcA- (A) and hgcB- (B) like sequences detected in the paired-end metagenomes from 500 m and 800 m depth at Sts. 0 and 1. Sequences identified in this study (shown in red) are compared with hgcAB homologues described in previous study (Gilmour et al., 2013; Gionfriddo et al., 2016; Gilmour et al., 2018; Christensen et al., 2019; Jones et al., 2019). Bootstrap values with consensus based on 100 replicates are shown. National Center for Biotechnology Information accession numbers or Integrated Microbial Genomes predicted gene numbers are provided.

## Slide 8
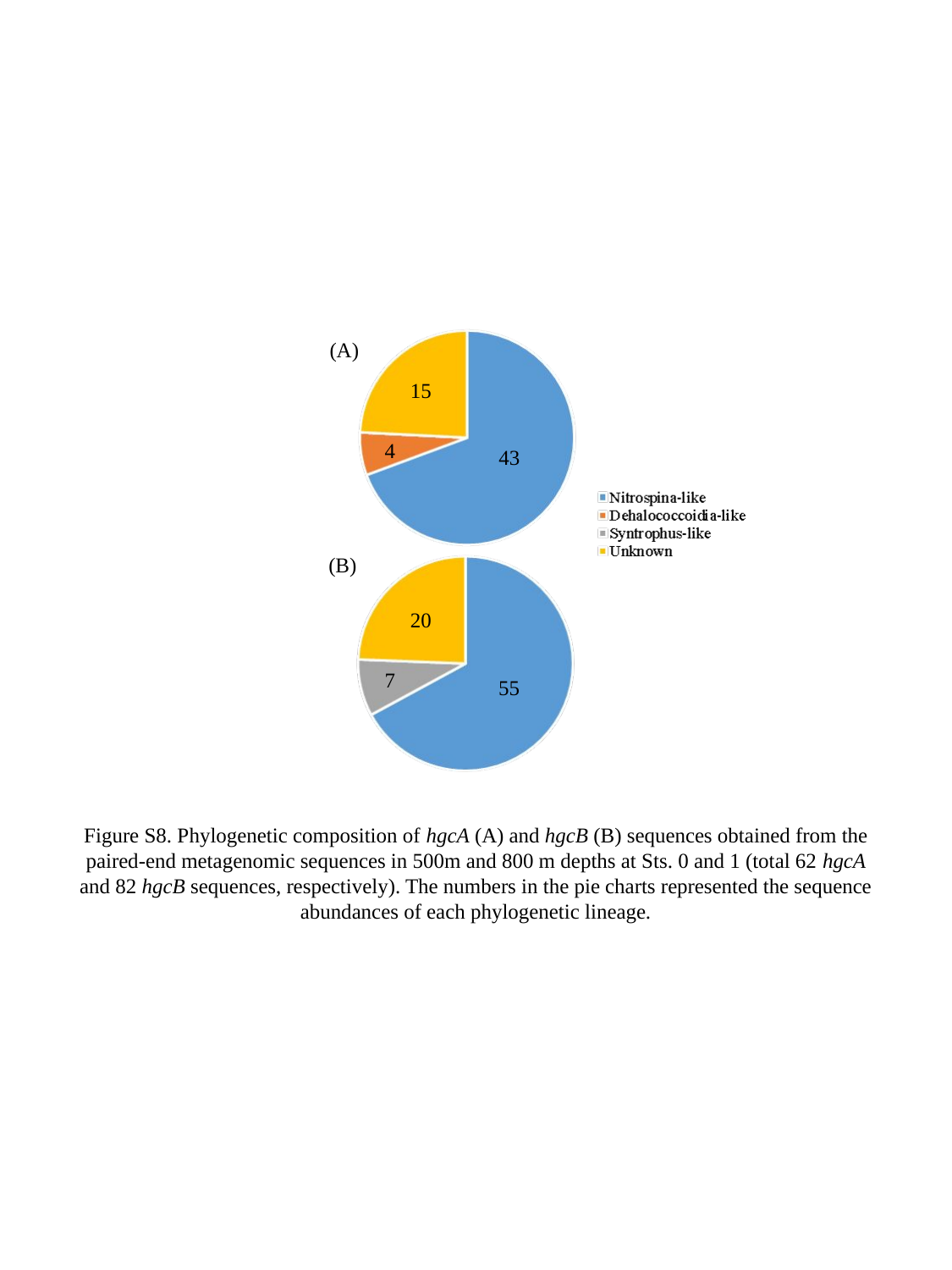

(A)
15
4
43
(B)
20
7
55
Figure S8. Phylogenetic composition of hgcA (A) and hgcB (B) sequences obtained from the paired-end metagenomic sequences in 500m and 800 m depths at Sts. 0 and 1 (total 62 hgcA and 82 hgcB sequences, respectively). The numbers in the pie charts represented the sequence abundances of each phylogenetic lineage.

## Slide 9
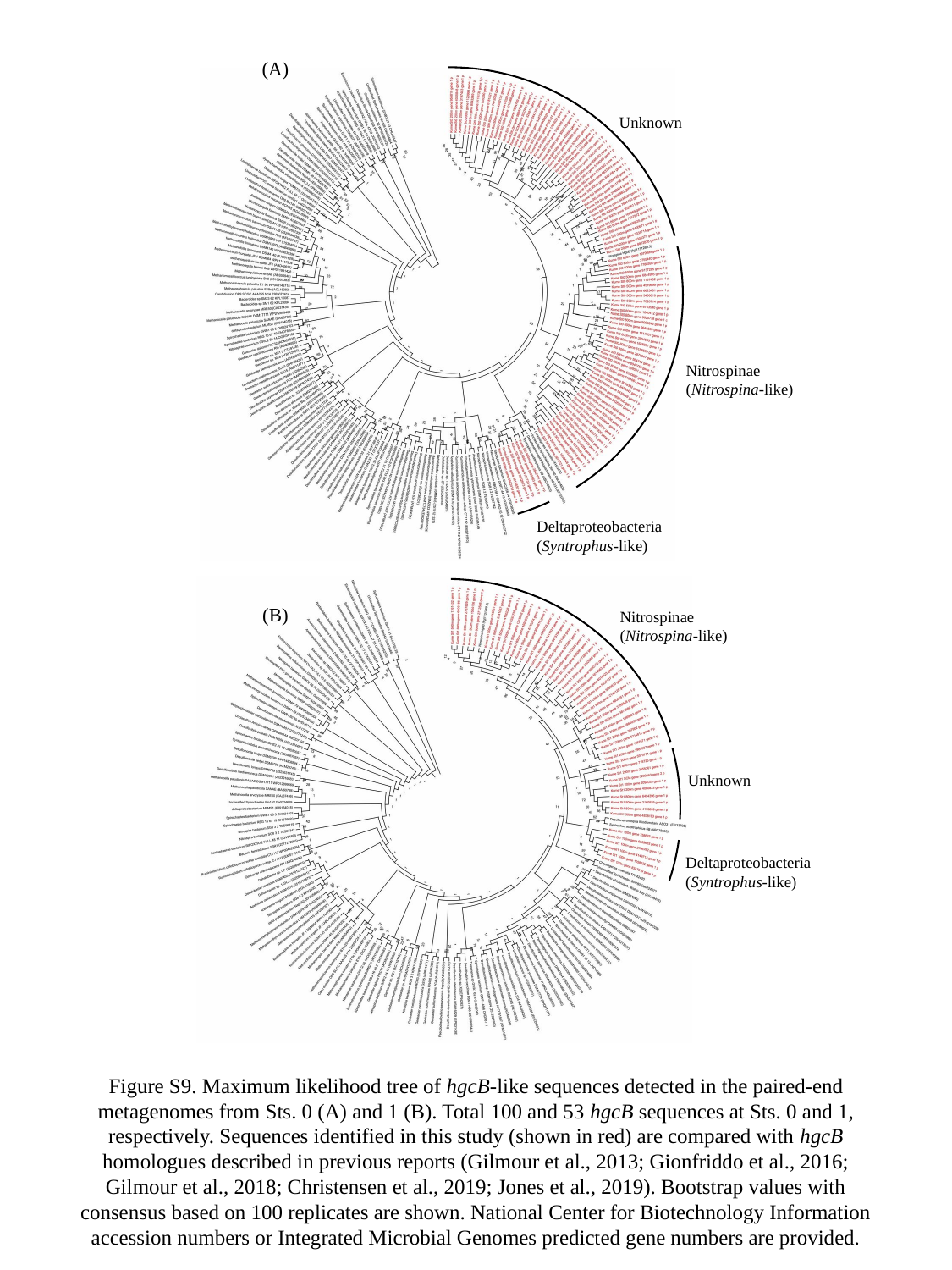

(A)
Unknown
Nitrospinae
(Nitrospina-like)
Deltaproteobacteria
(Syntrophus-like)
(B)
Nitrospinae
(Nitrospina-like)
Unknown
Deltaproteobacteria
(Syntrophus-like)
Figure S9. Maximum likelihood tree of hgcB-like sequences detected in the paired-end metagenomes from Sts. 0 (A) and 1 (B). Total 100 and 53 hgcB sequences at Sts. 0 and 1, respectively. Sequences identified in this study (shown in red) are compared with hgcB homologues described in previous reports (Gilmour et al., 2013; Gionfriddo et al., 2016; Gilmour et al., 2018; Christensen et al., 2019; Jones et al., 2019). Bootstrap values with consensus based on 100 replicates are shown. National Center for Biotechnology Information accession numbers or Integrated Microbial Genomes predicted gene numbers are provided.

## Slide 10
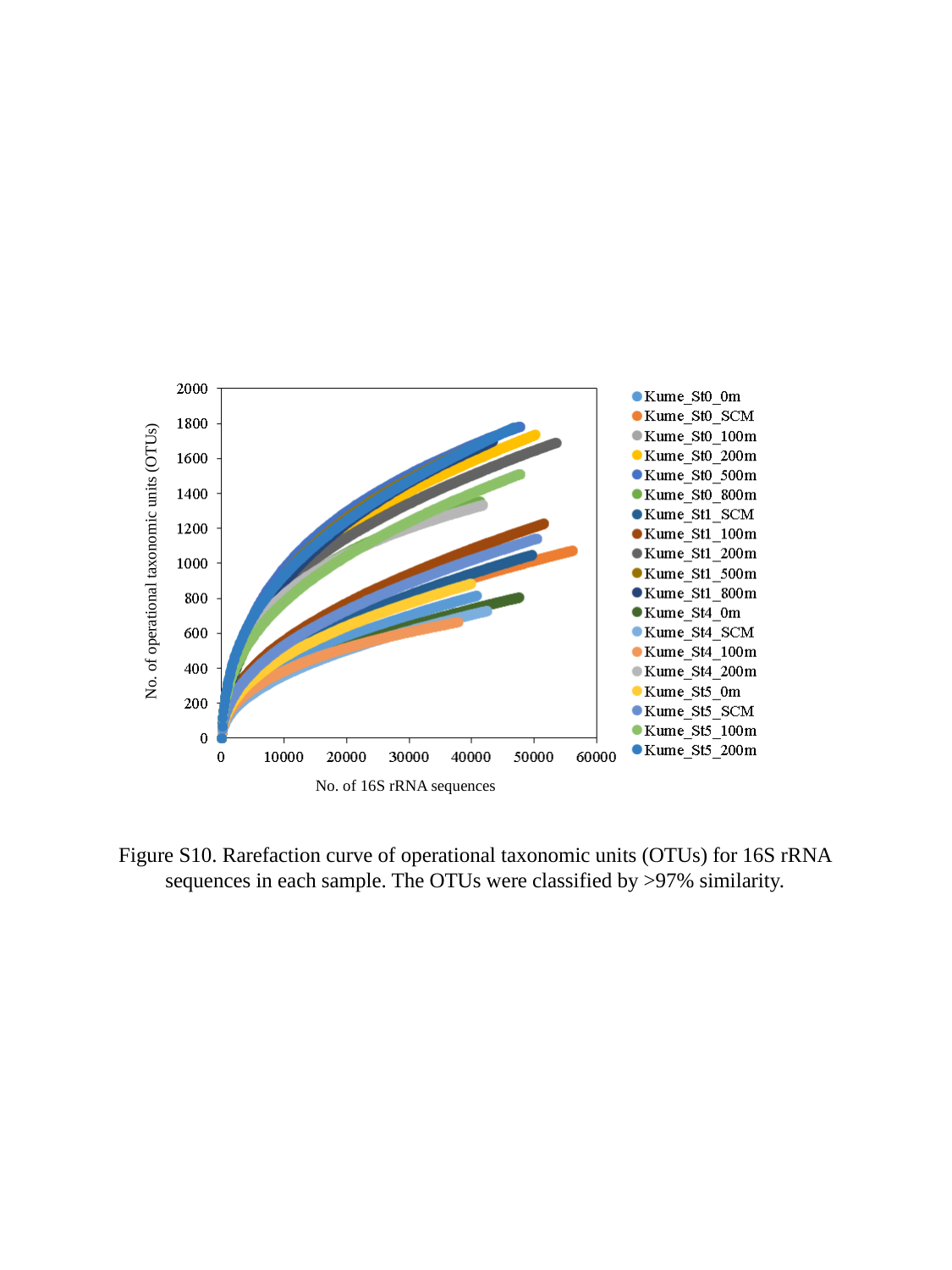

No. of operational taxonomic units (OTUs)
No. of 16S rRNA sequences
Figure S10. Rarefaction curve of operational taxonomic units (OTUs) for 16S rRNA sequences in each sample. The OTUs were classified by >97% similarity.

## Slide 11
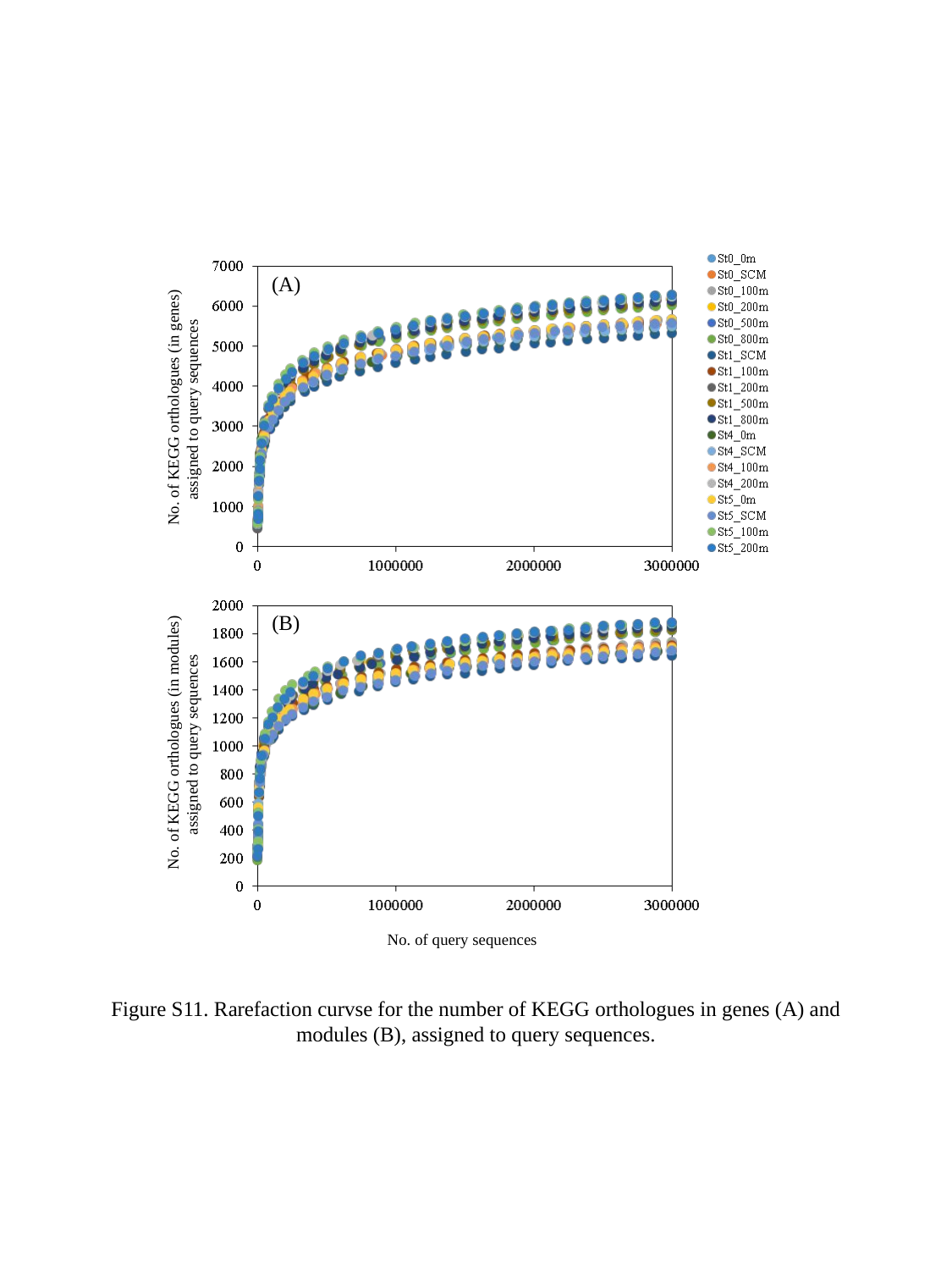

(A)
No. of KEGG orthologues (in genes) assigned to query sequences
(B)
No. of KEGG orthologues (in modules) assigned to query sequences
No. of query sequences
Figure S11. Rarefaction curvse for the number of KEGG orthologues in genes (A) and modules (B), assigned to query sequences.

## Slide 12
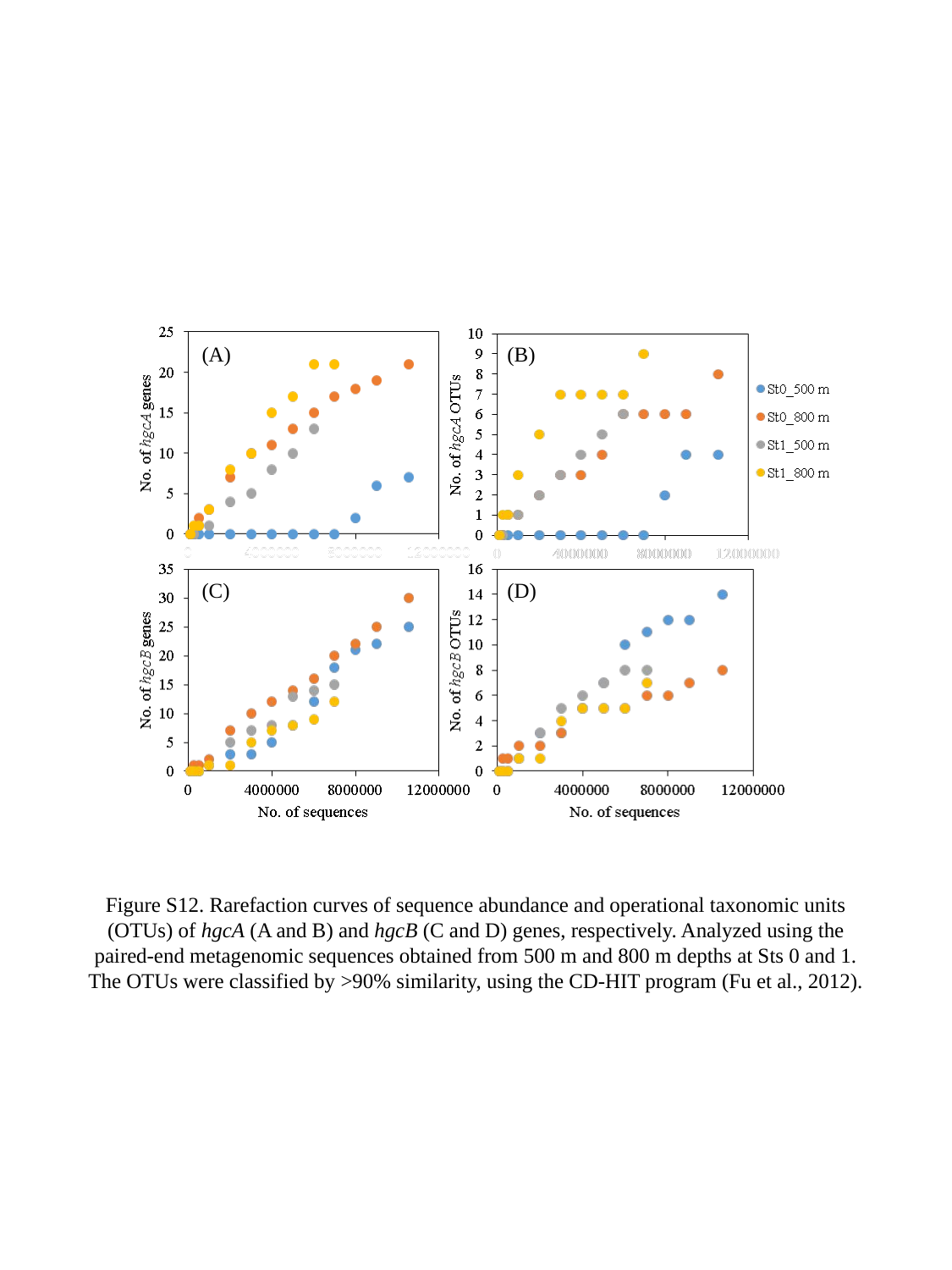

(A)
(B)
(C)
(D)
Figure S12. Rarefaction curves of sequence abundance and operational taxonomic units (OTUs) of hgcA (A and B) and hgcB (C and D) genes, respectively. Analyzed using the paired-end metagenomic sequences obtained from 500 m and 800 m depths at Sts 0 and 1. The OTUs were classified by >90% similarity, using the CD-HIT program (Fu et al., 2012).

## Slide 13
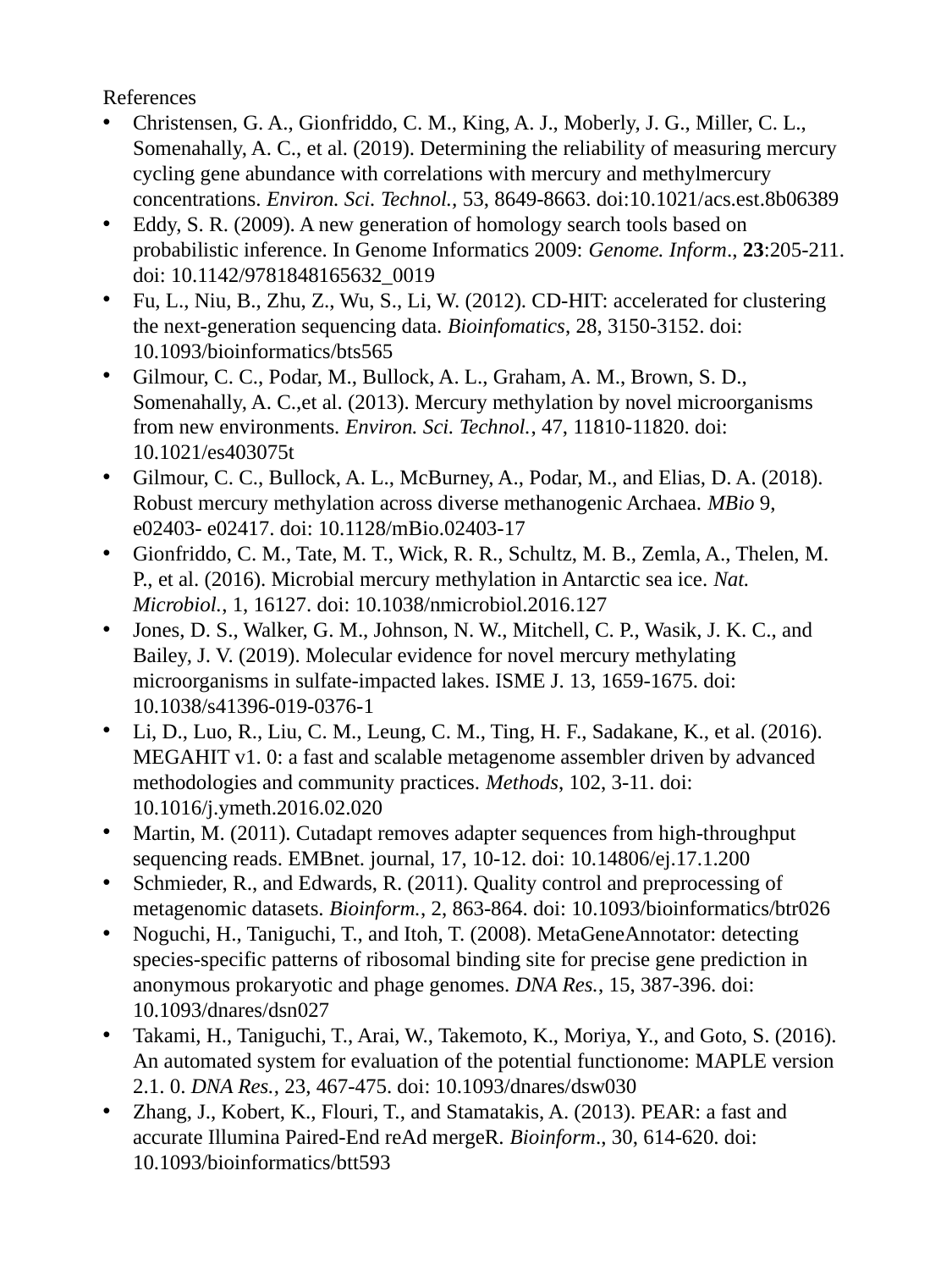

References
Christensen, G. A., Gionfriddo, C. M., King, A. J., Moberly, J. G., Miller, C. L., Somenahally, A. C., et al. (2019). Determining the reliability of measuring mercury cycling gene abundance with correlations with mercury and methylmercury concentrations. Environ. Sci. Technol., 53, 8649-8663. doi:10.1021/acs.est.8b06389
Eddy, S. R. (2009). A new generation of homology search tools based on probabilistic inference. In Genome Informatics 2009: Genome. Inform., 23:205-211. doi: 10.1142/9781848165632_0019
Fu, L., Niu, B., Zhu, Z., Wu, S., Li, W. (2012). CD-HIT: accelerated for clustering the next-generation sequencing data. Bioinfomatics, 28, 3150-3152. doi: 10.1093/bioinformatics/bts565
Gilmour, C. C., Podar, M., Bullock, A. L., Graham, A. M., Brown, S. D., Somenahally, A. C.,et al. (2013). Mercury methylation by novel microorganisms from new environments. Environ. Sci. Technol., 47, 11810-11820. doi: 10.1021/es403075t
Gilmour, C. C., Bullock, A. L., McBurney, A., Podar, M., and Elias, D. A. (2018). Robust mercury methylation across diverse methanogenic Archaea. MBio 9, e02403- e02417. doi: 10.1128/mBio.02403-17
Gionfriddo, C. M., Tate, M. T., Wick, R. R., Schultz, M. B., Zemla, A., Thelen, M. P., et al. (2016). Microbial mercury methylation in Antarctic sea ice. Nat. Microbiol., 1, 16127. doi: 10.1038/nmicrobiol.2016.127
Jones, D. S., Walker, G. M., Johnson, N. W., Mitchell, C. P., Wasik, J. K. C., and Bailey, J. V. (2019). Molecular evidence for novel mercury methylating microorganisms in sulfate-impacted lakes. ISME J. 13, 1659-1675. doi: 10.1038/s41396-019-0376-1
Li, D., Luo, R., Liu, C. M., Leung, C. M., Ting, H. F., Sadakane, K., et al. (2016). MEGAHIT v1. 0: a fast and scalable metagenome assembler driven by advanced methodologies and community practices. Methods, 102, 3-11. doi: 10.1016/j.ymeth.2016.02.020
Martin, M. (2011). Cutadapt removes adapter sequences from high-throughput sequencing reads. EMBnet. journal, 17, 10-12. doi: 10.14806/ej.17.1.200
Schmieder, R., and Edwards, R. (2011). Quality control and preprocessing of metagenomic datasets. Bioinform., 2, 863-864. doi: 10.1093/bioinformatics/btr026
Noguchi, H., Taniguchi, T., and Itoh, T. (2008). MetaGeneAnnotator: detecting species-specific patterns of ribosomal binding site for precise gene prediction in anonymous prokaryotic and phage genomes. DNA Res., 15, 387-396. doi: 10.1093/dnares/dsn027
Takami, H., Taniguchi, T., Arai, W., Takemoto, K., Moriya, Y., and Goto, S. (2016). An automated system for evaluation of the potential functionome: MAPLE version 2.1. 0. DNA Res., 23, 467-475. doi: 10.1093/dnares/dsw030
Zhang, J., Kobert, K., Flouri, T., and Stamatakis, A. (2013). PEAR: a fast and accurate Illumina Paired-End reAd mergeR. Bioinform., 30, 614-620. doi: 10.1093/bioinformatics/btt593
