# Supplementary material for: Nitrospina-Like Bacteria Are Potential Mercury Methylators in the Mesopelagic Zone in the East China Sea
Source: Front Microbiol. 2020 Jul 3;11:1369. doi: 10.3389/fmicb.2020.01369 (PMC7347909; doi:10.3389/fmicb.2020.01369)
Supplement: Supplementary file 4 [file Data_Sheet_3.pdf]

Table S1. Raw data of environmental factors

Station	Depth [m]	Remarks	THg (pM)	MeHg (pM)	MeHg / THg (%)	Seawater temperature (°C)	Salinity (PSU)	Dissolved oxygen (mg L^{-1})	Chlorophyll *a* (μg L^{-1})	Nitrite (μM)	Nitrate (μM)	Phosphate (μM)	Silicate (μM)	POC (μg L^{-1})	PN (μg L^{-1})	Prokaryotic cell abundance ($\times 10^5$ cells mL^{-1})
St.0	0		0.43	ND	ND	23.55	34.48	6.95	0.20	ND	0.06	ND	1.64	55.15	4.08	2.09
St.0	40	SCM	0.53	0.02	2.90	22.68	34.65	6.97	0.48	0.017	0.23	ND	1.53	55.01	4.29	1.74
St.0	100		0.57	ND	ND	22.30	34.81	7.11	0.26	0.029	0.21	ND	ND	46.96	2.88	1.57
St.0	200		0.47	0.01	1.30	20.52	34.94	6.39	0.05	0.039	1.50	0.10	2.12	34.34	0.46	0.55
St.0	300		0.53	0.02	3.91	18.00	34.82	6.43	0.05	0.017	4.82	0.29	4.54	NC	NC	NC
St.0	400		0.59	0.08	14.3	15.16	34.58	6.08	0.06	0.008	8.94	0.55	9.33	NC	NC	NC
St.0	500		0.72	0.19	26.8	12.58	34.40	5.61	0.07	ND	12.50	0.77	14.33	27.73	ND	0.29
St.0	600		1.00	0.49	48.8	8.19	34.27	3.80	0.10	ND	28.40	1.89	54.71	NC	NC	NC
St.0	700		0.98	0.42	43.1	6.50	34.29	3.04	0.13	ND	31.26	2.05	62.82	NC	NC	NC
St.0	800		1.04	0.62	59.4	6.09	34.29	2.89	0.12	ND	35.56	2.43	81.09	26.98	ND	0.28
St.1	0		1.05	0.00	0.44	23.94	34.41	6.93	0.28	ND	0.04	ND	1.54	NC	NC	NC
St.1	50	SCM	0.55	0.01	1.59	22.77	34.60	7.04	0.43	ND	0.06	ND	1.53	70.17	5.60	1.71
St.1	100		0.63	0.01	1.66	22.41	34.71	6.99	0.37	0.009	ND	ND	1.49	92.92	4.17	1.01
St.1	200		0.63	0.01	1.29	20.34	34.93	6.36	0.05	0.029	0.62	0.06	1.85	33.86	0.31	0.80
St.1	300		0.93	0.03	3.53	17.52	34.77	6.27	0.07	0.011	7.48	0.30	4.68	NC	NC	NC
St.1	400		0.69	0.12	16.8	13.95	34.49	6.12	0.06	0.014	8.68	0.53	8.62	NC	NC	NC
St.1	500		0.99	0.44	44.8	10.95	34.30	5.22	0.07	0.007	20.38	1.25	26.41	27.76	0.001	0.09
St.1	600		1.01	0.56	56.0	8.01	34.21	4.02	0.10	0.004	25.14	1.62	40.63	NC	NC	NC
St.1	700		1.01	0.73	71.9	6.70	34.29	3.14	0.12	ND	30.78	2.07	58.56	NC	NC	NC
St.1	800		0.92	0.51	55.2	5.72	34.31	2.75	0.13	0.004	35.39	2.46	81.85	28.39	ND	0.10
St.4	0		0.90	ND	ND	23.12	34.52	7.04	0.20	0.002	0.04	ND	1.58	56.09	4.54	1.57
St.4	40	SCM	0.48	ND	ND	21.94	34.51	7.21	0.74	ND	ND	ND	2.32	68.59	7.23	3.75
St.4	100		1.22	0.02	1.88	19.11	34.66	6.09	0.09	ND	1.99	0.18	3.79	78.17	9.54	2.75
St.4	200		0.68	0.08	11.8	13.63	34.50	5.43	0.07	0.031	9.49	0.61	13.25	42.62	1.20	0.37
St.5	0		0.74	ND	ND	22.46	34.61	7.09	0.23	ND	ND	ND	ND	54.07	4.07	1.66
St.5	40	SCM	0.46	ND	ND	22.55	34.79	7.08	0.26	ND	ND	ND	ND	50.24	3.14	1.55
St.5	100		0.57	ND	ND	22.46	34.88	7.04	0.24	ND	0.72	0.07	1.66	88.80	1.02	0.57
St.5	150		0.67	0.01	0.79	21.14	34.94	6.51	0.08	0.031	1.63	0.13	2.21	NC	NC	NC
St.5	200		0.77	0.01	0.77	19.60	34.86	6.46	0.06	0.019	2.55	0.18	2.69	33.08	0.32	0.24

ND: Not detected

NC: Not collected

Table S2. The list of *hgcA* genes for construction of the HMM reference

Strain	Phylum or class	Order	Accession no.	Length (amino acid)	References
Desulfobulbus japonicus DSM18378	Deltaproteobacteria	Desulfobacterales	2525725204	291	Gilmour et al., 2013
Desulfobulbus mediterraneus DSM13871	Deltaproteobacteria	Desulfobacterales	2523916689	323	
Desulfobulbus propionicus DSM2032	Deltaproteobacteria	Desulfobacterales	ADW16620	339	
Desulfococcus multivorans DSM2059	Deltaproteobacteria	Desulfobacterales	EPR42565	335	
Desulfospira joergensenii DSM10085	Deltaproteobacteria	Desulfobacterales	2523531012	399	
Desulfotignum balticum DSM7044	Deltaproteobacteria	Desulfobacterales	2526265153	402	
Desulfotignum phosphitoxidans DSM13687	Deltaproteobacteria	Desulfobacterales	EMS78346	402	
Desulfomicrobium baculatum DSM4028	Deltaproteobacteria	Desulfovibrionales	ACU88503	346	
Desulfonatronospira thiodismutans ASO31	Deltaproteobacteria	Desulfovibrionales	EFI33704	339	
Desulfonatronum lacustre Z7951 DSM10312	Deltaproteobacteria	Desulfovibrionales	2516164329	334	
Desulfovibrio africanus PCS	Deltaproteobacteria	Desulfovibrionales	EMG37847	325	
Desulfovibrio africanus str. Walvis Bay	Deltaproteobacteria	Desulfovibrionales	EGJ48477	325	
Desulfovibrio alkalitolerans DSM16529	Deltaproteobacteria	Desulfovibrionales	EPR31439	347	
Desulfovibrio desulfuricans ND132	Deltaproteobacteria	Desulfovibrionales	EGB14269	338	
Desulfovibrio inopinatus DSM10711	Deltaproteobacteria	Desulfovibrionales	2525213991	308	
Desulfovibrio longus DSM6739	Deltaproteobacteria	Desulfovibrionales	2523631744	344	
Desulfovibrio oxyclinae DSM11498	Deltaproteobacteria	Desulfovibrionales	2515862843	332	
Desulfovibrio putealis DSM16056	Deltaproteobacteria	Desulfovibrionales	2523324479	336	
Desulfovibrio sp. X2	Deltaproteobacteria	Desulfovibrionales	EPR42826	349	
Pseudodesulfovibrio aespoeensis Aspo2	Deltaproteobacteria	Desulfovibrionales	ADU63658	334	
Geobacter bemidjiensis Bem	Deltaproteobacteria	Geobacteraceae	ACH38202	322	
Geobacter daltonii FRC32	Deltaproteobacteria	Geobacteraceae	ACM20836	324	
Geobacter metallireducens GS15	Deltaproteobacteria	Geobacteraceae	ABB31476	323	
Geobacter metallireducens RCH3	Deltaproteobacteria	Geobacteraceae	EHP88436	323	
Geobacter sp. M18	Deltaproteobacteria	Geobacteraceae	ADW12506	319	
Geobacter sp. M21	Deltaproteobacteria	Geobacteraceae	ACT19119	322	
Geobacter sulfurreducens KN400	Deltaproteobacteria	Geobacteraceae	ADI84278	325	
Geobacter sulfurreducens PCA	Deltaproteobacteria	Geobacteraceae	AAR34814	325	
Geobacter uraniireducens Rf4	Deltaproteobacteria	Geobacteraceae	ABQ24695	318	
Desulfomonile tiedjei DSM6799	Deltaproteobacteria	Syntrophobacterales	AFM23739	339	
Syntrophorhabdus aromaticivorans UI	Deltaproteobacteria	Syntrophobacterales	2509867054	347	
Syntrophus aciditrophicus SB	Deltaproteobacteria	Syntrophobacterales	ABC78804	396	
Geopsychrobacter electrodiphilus DSM16401	Deltaproteobacteria	Desulfuromonadales	2522771244	394	
Delta proteobacterium MLMS1	Deltaproteobacteria		639154017	317	
Delta proteobacterium NaphS2	Deltaproteobacteria		EFK05849	353	
Acetivibrio cellulolyticus CD2 DSM1870	Firmicutes	Clostridiales	2510774674	401	
Dehalobacter restrictus DSM9455	Firmicutes	Clostridiales	2510121322	385	
Dehalobacter sp.	Firmicutes	Clostridiales	2520069246	385	
Dehalobacter sp. 11DCA	Firmicutes	Clostridiales	2520954802	385	
Desulfitobacterium dehalogenans ATCC51507	Firmicutes	Clostridiales	AFM01083	334	
Desulfitobacterium dichloroeliminans LMG P21439	Firmicutes	Clostridiales	AGA68305	334	
Desulfitobacterium metallireducens DSM15288	Firmicutes	Clostridiales	EHC09800	334	
Desulfitobacterium sp. PCE1 DSM10344	Firmicutes	Clostridiales	2512921981	334	
Desulfosporosinus acidiphilus SJ4	Firmicutes	Clostridiales	AFM40623	334	
Desulfosporosinus orientis DSM765	Firmicutes	Clostridiales	AET68199	334	
Desulfosporosinus sp. OT	Firmicutes	Clostridiales	EGW36302	335	
Desulfosporosinus youngiae DSM17734	Firmicutes	Clostridiales	EHQ91195	336	
Dethiobacter alkaliphilus AHT1	Firmicutes	Clostridiales	EEG75985	373	
Ethanoligenens harbinense YUAN3	Firmicutes	Clostridiales	ADU26528	369	
Ruminiclostridium cellobioparum subsp. termitidis CT1112	Firmicutes	Clostridiales	EMS71460	404	
Syntrophobotulus glycolicus DSM8271	Firmicutes	Clostridiales	ADY56639	352	
Acetonema longum DSM 6540	Firmicutes	Selenomonadales	EGO62461	380	
Dehalococcoides mccartyi DCMB5	Chloroflexi	Dehalococcoidales	AGG05961	341	
Bacteria ferrireducans S3R1	Bacteria		2517272092	403	
Methanoregula boonei 6A8	Euryarchaeota	Methanomicrobiales	ABS54941	345	
Methanoregula formicica SMSP	Euryarchaeota	Methanomicrobiales	AGB02006	359	
Methanosphaerula palustris E19c	Euryarchaeota	Methanomicrobiales	ACL16382	320	
Methanospirillum hungatei JF1	Euryarchaeota	Methanomicrobiales	ABD40626	342	
Methanocorpusculum bavaricum DSM4179	Euryarchaeota	Methanomicrobiales	2525334638	356	
Methanofollis liminatans DSM4140	Euryarchaeota	Methanomicrobiales	EJG07630	342	
Methanocella arvoryzae MRE50	Euryarchaeota	Methanocellales	CAJ37437	332	
Methanocella paludicola SANAE	Euryarchaeota	Methanocellales	BAI60790	331	
Methanomethylovorans hollandica DSM15978	Euryarchaeota	Methanosarcinales	AGB48887	354	
Methanolobus psychrophilus R15	Euryarchaeota	Methanosarcinales	AFV22798	356	
Methanomassiliicoccus luminyensis B10	Euryarchaeota	Methanomassiliicoccales	2518907384	324	
Marine benthic group B archaeon SG881	Euryarchaeota		KON27549	547	Gionfriddo et al., 2016
Kosmotoga pacifica	Kosmotoga	Kosmotogales	WP047755538	384	
Methanococcoides methylutens	Euryarchaeota	Methanosarcinales	WP048193041	383	
Pyrococcus furiosus DSM3638	Euryarchaeota	Thermococcales	NC003413	388	
Nitrospina sp.	Nitrospina		WP017952925	289	

Bacteroides_sp_SM1_62	Bacteroidetes	Bacteroidetes	KPL23093	328	Christensen et al., 2019
Bacteroides_sp_SM23_62	Bacteroidetes	Bacteroidetes	KPL16088	328	
Bacteroidetes_bacterium_GWA2_32_17	Bacteroidetes	Bacteroidetes	OFX29274	338	
Bacteroidetes_bacterium_GWF2_35_48	Bacteroidetes	Bacteroidetes	OFY38355	342	
Bacteroidetes_bacterium_HGW_Bacteroidetes_21	Bacteroidetes	Bacteroidetes	PKP19910	343	
Bacteroidetes_bacterium_RBG_13_46_8	Bacteroidetes	Bacteroidetes	OFY57839	344	
Bacteroidetes_bacterium_RIFOXYA12_FULL_35_11	Bacteroidetes	Bacteroidetes	OFY82703	342	
Bacteroidetes_cellulosolvens_DSM2933	Bacteroidetes	Bacteroidetes	KNY29400	378	
Cand_division_OP9_SCGC_AAA255_N14	Cand. Division OP9		2265072415	331	
Dehalococcoidia_bacterium_SCGC_AG_205_B13	Chloroflexi	Dehalococcoidales	Ga0157251	389	
Dehalococcoidia_bacterium_SCGC_AG_205_I02	Chloroflexi	Dehalococcoidales	Ga0157268	389	
Dehalococcoidia_bacterium_SCGC_AG_205_I13	Chloroflexi	Dehalococcoidales	Ga0153886	389	
Chrysiogenes_arsenatis	Chrysiogenes	Chrysiogenales	WP152514490	321	
Bdellovibrionales_bacterium_RIFOXYB1_FULL_39_21	Oligoflexia		OFZ38049	374	
Bdellovibrionales_bacterium_RIFOXYC1_FULL_39_130	Oligoflexia		OFZ48784	374	
Bdellovibrionales_bacterium_RIFOXYC1_FULL_54_43	Oligoflexia		OFZ55613	385	
Bdellovibrionales_bacterium_RIFOXYC12_FULL_39_17	Oligoflexia		OFZ43882	374	
Desulfomonile_tiedjei_DSM6799	Deltaproteobacteria	Syntrophobacterales	WP041285976	348	
Desulfobacula_phenolica_DSM3384	Deltaproteobacteria	Desulfobacterales	WP092232717	397	
Desulfuromonas_soudanensis	Deltaproteobacteria	Desulfuromonadales	ALC17221	376	
Desulfonatronum_thiosulfatophilum	Deltaproteobacteria	Desulfovibrionales	SDB24809	181	
Elusimicrobia_bacterium_RIFOXYA2_FULL_40_6	Elusimicrobia		OGS20981	316	
Elusimicrobia_bacterium_RIFOXYA2_FULL_47_53	Elusimicrobia		OGS16464	370	
Elusimicrobia_bacterium_RIFOXYA12_FULL_49_49	Elusimicrobia		OGS02014	370	
Elusimicrobia_bacterium_RIFOXYB1_FULL_48_9	Elusimicrobia		OGS10053	370	
Elusimicrobia_bacterium_RIFOXYB2_FULL_46_23	Elusimicrobia		OGS29648	370	
Elusimicrobia_bacterium_RIFOXYB2_FULL_48_7	Elusimicrobia		OGS27984	277	
Elusimicrobia_bacterium_RIFOXYB12_FULL_50_12	Elusimicrobia		OGS26031	370	
Methanoregula_boonei_6A8	Euryarchaeota	Methanomicrobia	WP011991429	345	
Methanoregula_formicica_SMSP	Euryarchaeota	Methanomicrobia	AGB02006	359	
Clostridium_litorale_DSM5388	Firmicutes	Clostridiales	KDR96580	359	
Clostridium_litorale_DSM5388	Firmicutes	Clostridiales	WP161694518	312	
Clostridium_litorale_DSM5388	Firmicutes	Clostridiales	WP159434243	353	
Clostridium_litorale_DSM5388	Firmicutes	Clostridiales	SIN68872	359	
Clostridium_tunisiense_TJ	Firmicutes	Clostridiales	WP017416929	364	
Anaerocolumna_jejuensis_DSM15929	Firmicutes	Clostridiales	SHK87540	368	
Anaerocolumna_xylanovorans_DSM12503	Firmicutes	Clostridiales	SHO54149	368	
Ruminiclostridium_cellobioparum_subsp_termitidis_CT1112	Firmicutes	Clostridiales	WP004626173	404	
Lentisphaerae_bacterium_RIFOXYA12_FULL_48_11	Lentisphaerae		OGV64895	328	
Nitrospira_bacterium_SG8_3_2	Nitrospira		KPK34762	343	
Nitrospira_bacterium_SG8_3_2	Nitrospira		KPK26723	342	
Nitrospira_bacterium_SG8_3_2	Nitrospira		TES56118	359	
Nitrospira_bacterium_SG8_3_2	Nitrospira		KPK25308	343	
Nitrospira_bacterium_SG8_3	Nitrospira		KPK26723	342	
Nitrospira_bacterium_SG8_3	Nitrospira		KPK25308	343	
Nitrospirae_bacterium_GWC2_56_14	Nitrospira		OGW38354	360	
Nitrospirae_bacterium_GWC2_56_14	Nitrospira		OGW34114	321	
Nitrospirae_bacterium_GWF2_44_13	Nitrospira		OGW30936	344	
Nitrospirae_bacterium_RBG_19FT_COMBO_55_12	Nitrospira		OGW52723	341	
Spirochaetes_bacterium_GWB1_27_13	Spirochaetes		OHD16550	335	
Spirochaetes_bacterium_GWB1_36_13	Spirochaetes		OHD14484	335	
Spirochaetes_bacterium_GWB1_60_80	Spirochaetes		OHD18750	336	
Spirochaetes_bacterium_GWE2_31_10	Spirochaetes		OHD50409	342	
Spirochaetes_bacterium_GWF1_31_7	Spirochaetes		OHD52498	342	
Spirochaetes_bacterium_GWF1_60_12	Spirochaetes		OHD58568	336	
Spirochaetes_bacterium_RBG_16_49_21	Spirochaetes		OHD69644	349	
Spirochaetes_bacterium_RBG_16_67_19	Spirochaetes		OHD76042	358	
Spirochaetes_bacterium_RIFOXYB1_FULL_32_8	Spirochaetes		OHD81691	342	
Spirochaetes_bacterium_GWF1_49_6	Spirochaetes		OHD58719	333	
Spirochaetes_bacterium_GWF1_51_8	Spirochaetes		OHD55128	343	
Spirochaetes_bacterium_GWB1_66_5	Spirochaetes		OHD24105	342	
Treponema_sp_GWA1_62_8	Spirochaetes	Spirochaetales	OHE66044	357	
Alkalispirochaeta_odontotermitis	Spirochaetes	Spirochaetales	WP037564355	331	
Unclassified_Spirochaetes_Bin043	Spirochaetes		Ga0224667	360	Jones et al., 2019
Unclassified_Aminicenantes_OP8_Bin144	Cand. Aminicenantes		Ga0207195	371	
Unclassified_Spirochaetes_Bin152	Spirochaetes		Ga0224669	354	
Unclassified_Spirochaetes_Bin159	Spirochaetes		Ga0224664	332	
Unclassified_Spirochaetes_Bin180	Spirochaetes		Ga0224672	335	
Unclassified_PVC_group_bacterium_Bin55r1	Unclassified PVC group		Ga0224692	346	
Methanofollis_liminatans_DSM4140	Euryarchaeota	Methanomicrobia	WP157203321	294	Gilmour et al., 2018
Methanosphaerula_palustris_E1_9c	Euryarchaeota	Methanomicrobia	WP148208277	339	
Methanocella_paludicola_SANAE_DSM17711	Euryarchaeota	Methanomicrobia	WP012899470	331	
Methanocorpusculum_bavaricum_DSM4179	Euryarchaeota	Methanomicrobia	WP042697303	356	
Methanomethylovorans_hollandica_DSM15978	Euryarchaeota	Methanomicrobia	WP015324055	354	
Methanospirillum_hungatei_JF_1_DSM864	Euryarchaeota	Methanomicrobia	WP143709353	348	

Table S3. The list of *hgcB* genes for construction of the HMM reference

Strain	Phylum or class	Order	Accession no.	Length (amino acid)	References
Desulfobulbus japonicus DSM18378	Deltaproteobacteria	Desulfobacterales	2525725205	95	Gilmour et al., 2013
Desulfobulbus mediterraneus DSM13871	Deltaproteobacteria	Desulfobacterales	2523916683	96	
Desulfobulbus propionicus DSM2032	Deltaproteobacteria	Desulfobacterales	ADW16619	95	
Desulfococcus multivorans DSM2059	Deltaproteobacteria	Desulfobacterales	EPR42566	94	
Desulfotignum balticum DSM7044	Deltaproteobacteria	Desulfobacterales	2526265152	72	
Desulfotignum phosphitoxidans DSM13687	Deltaproteobacteria	Desulfobacterales	EMS78347	72	
Desulfospira joergensenii DSM10085	Deltaproteobacteria	Desulfobacterales	2523531013	72	
Desulfomicrobium baculatum DSM4028	Deltaproteobacteria	Desulfovibrionales	ACU88502	91	
Desulfovibrio africanus PCS	Deltaproteobacteria	Desulfovibrionales	EMG37849	96	
Desulfovibrio africanus str. Walvis Bay	Deltaproteobacteria	Desulfovibrionales	EGJ48475	96	
Desulfovibrio alkalitolerans DSM16529	Deltaproteobacteria	Desulfovibrionales	EPR31438	99	
Desulfovibrio desulfuricans ND132	Deltaproteobacteria	Desulfovibrionales	EGB14270	95	
Desulfovibrio inopinatus DSM10711	Deltaproteobacteria	Desulfovibrionales	2525213937	93	
Desulfovibrio longus DSM6739	Deltaproteobacteria	Desulfovibrionales	2523631745	92	
Desulfovibrio oxyclinae DSM11498	Deltaproteobacteria	Desulfovibrionales	2515862844	91	
Desulfovibrio putealis DSM16056	Deltaproteobacteria	Desulfovibrionales	2523324480	100	
Desulfovibrio sp. X2	Deltaproteobacteria	Desulfovibrionales	EPR42827	100	
Pseudodesulfovibrio aespoeensis Aspo2	Deltaproteobacteria	Desulfovibrionales	ADU63659	95	
Desulfonatronospira thiodismutans ASO31	Deltaproteobacteria	Desulfovibrionales	EFI33705	104	
Desulfonatronum lacustre Z7951 DSM10312	Deltaproteobacteria	Desulfovibrionales	2516164328	91	
Geobacter bemidjiensis Bem	Deltaproteobacteria	Geobacteraceae	ACH38203	97	
Geobacter daltonii FRC32	Deltaproteobacteria	Geobacteraceae	ACM20835	95	
Geobacter metallireducens GS15	Deltaproteobacteria	Geobacteraceae	ABB31477	96	
Geobacter metallireducens RCH3	Deltaproteobacteria	Geobacteraceae	EHP88435	96	
Geobacter sp. M18	Deltaproteobacteria	Geobacteraceae	ADW12507	97	
Geobacter sp. M21	Deltaproteobacteria	Geobacteraceae	ACT19118	97	
Geobacter sulfurreducens KN400	Deltaproteobacteria	Geobacteraceae	ADI84280	94	
Geobacter sulfurreducens PCA	Deltaproteobacteria	Geobacteraceae	AAR34815	94	
Geobacter uraniireducens Rf4	Deltaproteobacteria	Geobacteraceae	ABQ24696	96	
Desulfomonile tiedjei DSM6799	Deltaproteobacteria	Syntrophobacterales	AFM23740	111	
Syntrophorhabdus aromaticivorans	Deltaproteobacteria	Syntrophobacterales	2509867055	105	
Syntrophus aciditrophicus SB	Deltaproteobacteria	Syntrophobacterales	ABC78805	81	
Geopsychrobacter electrodiphilus DSM16401	Deltaproteobacteria	Desulfuromonadales	2522771243	97	
Delta proteobacterium MLMS1	Deltaproteobacteria		639154018	93	
Delta proteobacterium NaphS2	Deltaproteobacteria		EFK05860	109	
Acetivibrio cellulolyticus DSM1870	Firmicutes	Clostridiales	2510774675	100	
Dehalobacter restrictus DSM9455	Firmicutes	Clostridiales	2510121321	99	
Dehalobacter sp.	Firmicutes	Clostridiales	2520069245	99	
Dehalobacter sp. 11DCA	Firmicutes	Clostridiales	2520954801	99	
Desulfitobacterium dehalogenans ATCC51507	Firmicutes	Clostridiales	AFM01082	98	
Desulfitobacterium dichloroeliminans	Firmicutes	Clostridiales	AGA68306	98	
Desulfitobacterium metallireducens DSM15288	Firmicutes	Clostridiales	EHC09801	96	
Desulfitobacterium sp. DSM10344	Firmicutes	Clostridiales	2512921980	98	
Desulfosporosinus acidiphilus SJ4	Firmicutes	Clostridiales	AFM40624	96	
Desulfosporosinus orientis DSM765	Firmicutes	Clostridiales	AET68200	96	
Desulfosporosinus sp.	Firmicutes	Clostridiales	EGW36301	100	
Desulfosporosinus youngiae DSM17734	Firmicutes	Clostridiales	EHQ91194	99	
Dethiobacter alkaliphilus AHT1	Firmicutes	Clostridiales	EEG75984	71	
Ethanoligenens harbinense YUAN3	Firmicutes	Clostridiales	ADU26529	98	
Ruminiclostridium cellobioparum subsp. CT1112	Firmicutes	Clostridiales	EMS71510	100	
Syntrophobotulus glycolicus DSM8271	Firmicutes	Clostridiales	ADY56638	97	
Acetonema longum DSM6540	Firmicutes	Selenomonadales	EGO62460	101	
Dehalococcoides mccartyi DCMB5	Chloroflexi	Dehalococcoidales	AGG05960	123	
Bacteria ferrireducans S3R1	Bacteria		2517272093	97	
Methanoregula boonei 6A8	Euryarchaeota	Methanomicrobiales	ABS54940	118	
Methanoregula formicica SMSP	Euryarchaeota	Methanomicrobiales	AGB02007	103	
Methanosphaerula palustris E19c	Euryarchaeota	Methanomicrobiales	ACL16383	113	
Methanospirillum hungatei JF1	Euryarchaeota	Methanomicrobiales	ABD40625	102	
Methanocorpusculum bavaricum DSM4179	Euryarchaeota	Methanomicrobiales	2525334639	116	
Methanofollis liminatans DSM4140	Euryarchaeota	Methanomicrobiales	EJG07629	110	
Methanocella arvoryzae MRE50	Euryarchaeota	Methanocellales	CAJ37436	118	
Methanocella paludicola SANAE	Euryarchaeota	Methanocellales	BAI60789	100	
Methanomethylovorans hollandica DSM15978	Euryarchaeota	Methanosarcinales	AGB48886	99	
Methanolobus psychrophilus R15	Euryarchaeota	Methanosarcinales	AFV22797	118	
Methanomassiliicoccus luminyensis B10	Euryarchaeota	Methanomassiliicoccales	2518907383	97	
Nitrospina sp.	Nitrospina		1131269.3	162	Gionfriddo et al., 2016

Bacteroides_sp_SM1_62	KPL23094	Bacteroidetes	Bacteroidetes	90	Christensen et al., 2019
Bacteroides_sp_SM23_62	KPL16087	Bacteroidetes	Bacteroidetes	90	
Bacteroidetes_bacterium_GWA2_32_17	OFX28371	Bacteroidetes	Bacteroidetes	97	
Bacteroidetes_bacterium_GWF2_35_48	OFY38354	Bacteroidetes	Bacteroidetes	99	
Bacteroidetes_bacterium_HGW_Bacteroidetes_21	PKP19919	Bacteroidetes	Bacteroidetes	89	
Bacteroidetes_bacterium_RIFOXYA12_FULL_35_11	OFY82702	Bacteroidetes	Bacteroidetes	99	
Bacteroidetes_cellulosolvens_DSM2933	WP036935625	Bacteroidetes	Bacteroidetes	99	
Cand_division_OP9_SCGC_AAA255_N14	2265072414	Cand. Division OP9		99	
Chrysiogenes_arsenatis	TFH42420	Chrysiogenes	Chrysiogenales	91	
Desulfomonile_tiedjei_DSM6799	WP014808894	Deltaproteobacteria	Syntrophobacterales	111	
Desulfobacula_phenolica_DSM3384	WP092232719	Deltaproteobacteria	Desulfobacterales	72	
Desulfuromonas_soudanensis	ALC17222	Deltaproteobacteria	Desulfuromonadales	94	
Desulfonatronum_thiosulfatophilum	SDB24847	Deltaproteobacteria	Desulfovibrionales	90	
Elusimicrobia_bacterium_RIFOXYA2_FULL_40_6	OGS20980	Elusimicrobia		98	
Elusimicrobia_bacterium_RIFOXYA2_FULL_47_53	OGS16463	Elusimicrobia		88	
Elusimicrobia_bacterium_RIFOXYA12_FULL_49_49	OGS02015	Elusimicrobia		88	
Elusimicrobia_bacterium_RIFOXYB1_FULL_48_9	OGS10054	Elusimicrobia		88	
Elusimicrobia_bacterium_RIFOXYB2_FULL_46_23	OGS29649	Elusimicrobia		88	
Elusimicrobia_bacterium_RIFOXYB12_FULL_50_12	OGS26032	Elusimicrobia		88	
Methanoregula_boonei_6A8	WP011991428	Euryarchaeota	Methanomicrobia	118	
Methanoregula_formicica_SMSP	AGB02007	Euryarchaeota	Methanomicrobia	103	
Clostridium_litorale_DSM5388	WP038261074	Firmicutes	Clostridiales	69	
Clostridium_tunisiense_TJ	WP083861720	Firmicutes	Clostridiales	94	
Anaerocolumna_jejuensis_DSM15929	SHK87576	Firmicutes	Clostridiales	97	
Anaerocolumna_xylanovorans_DSM12503	SHO54148	Firmicutes	Clostridiales	97	
Ruminiclostridium_cellobioparum_subsp_termitidis_CT1112	WP004626264	Firmicutes	Clostridiales	100	
Lentisphaerae_bacterium_RIFOXYA12_FULL_48_11	OGV64894	Lentisphaerae		94	
Nitrospira_bacterium_SG8_3_2	KPK34758	Nitrospira		92	
Nitrospira_bacterium_SG8_3_2	KPK25307	Nitrospira		115	
Nitrospira_bacterium_SG8_3_2	TES87342	Nitrospira		100	
Nitrospira_bacterium_SG8_3_2	TES56119	Nitrospira		96	
Nitrospira_bacterium_SG8_3	KPK25307	Nitrospira		115	
Nitrospirae_bacterium_GWC2_56_14	OGW38355	Nitrospira		97	
Nitrospirae_bacterium_GWC2_56_14	OGW34102	Nitrospira		96	
Nitrospirae_bacterium_GWF2_44_13	OGW30685	Nitrospira		98	
Nitrospirae_bacterium_RBG_19FT_COMBO_55_12	OGW52722	Nitrospira		98	
Spirochaetes_bacterium_GWB1_27_13	OHD16547	Nitrospira		110	
Spirochaetes_bacterium_GWB1_36_13	OHD14466	Spirochaetes		96	
Spirochaetes_bacterium_GWB1_60_80	OHD18715	Spirochaetes		100	
Spirochaetes_bacterium_GWE2_31_10	OHD50057	Spirochaetes		96	
Spirochaetes_bacterium_GWF1_31_7	OHD52371	Spirochaetes		96	
Spirochaetes_bacterium_GWF1_60_12	OHD58564	Spirochaetes		100	
Spirochaetes_bacterium_RBG_16_49_21	OHD69583	Spirochaetes		89	
Spirochaetes_bacterium_RBG_16_67_19	OHD76020	Spirochaetes		99	
Spirochaetes_bacterium_RIFOXYB1_FULL_32_8	OHD81687	Spirochaetes		96	
Spirochaetes_bacterium_GWF1_49_6	OHD58711	Spirochaetes		99	
Spirochaetes_bacterium_GWF1_51_8	OHD55129	Spirochaetes		129	
Spirochaetes_bacterium_GWB1_66_5	OHD24103	Spirochaetes		99	
Treponema_sp_GWA1_62_8	OHE66040	Spirochaetes		120	
Alkalispirochaeta_odontotermitis	WP037564333	Spirochaetes	Spirochaetales	96	
Unclassified_Spirochaetes_Bin043	Ga0224667	Spirochaetes	Spirochaetales	105	Jones et al., 2019
Unclassified_Aminicenantes_OP8_Bin144	Ga0207195	Cand. Aminicenantes		102	
Unclassified_Spirochaetes_Bin152	Ga0224669	Spirochaetes		97	
Unclassified_Spirochaetes_Bin159	Ga0224664	Spirochaetes		100	
Unclassified_Spirochaetes_Bin180	Ga0224672	Spirochaetes		91	
Unclassified_PVC_group_bacterium_Bin55r1	Ga0224692	Unclassified PVC group		94	
Methanofollis_liminatans_DSM4140	WP004039396	Euryarchaeota	Methanomicrobia	110	Gilmour et al., 2018
Methanosphaerula_palustris_E1_9c	WP048145718	Euryarchaeota	Methanomicrobia	112	
Methanocella_paludicola_SANAE_DSM17711	WP012899469	Euryarchaeota	Methanomicrobia	100	
Methanocorpusculum_bavaricum_DSM4179	WP042697304	Euryarchaeota	Methanomicrobia	116	
Methanomethylovorans_hollandica_DSM15978	WP015324054	Euryarchaeota	Methanomicrobia	99	
Methanospirillum_hungatei_JF_1_DSM864	WP011447904	Euryarchaeota	Methanomicrobia	102	

Table S4. Relative abundance of the prokaryotic lineages (16S rRNA gene amplicon analysis with QIIME v. 1.7.0)

Phylogenetic lineages	Percentage of total 16S rRNA gene amplicon sequences (%)																		
	St. 0						St. 1					St. 4				St. 5			
	0 m	SCM	100 m	200 m	500 m	800 m	SCM	100 m	200 m	500 m	800m	0 m	SCM	100 m	200 m	0 m	SCM	100 m	200 m
Euryarchaeota	1.2	0.7	1.4	4.6	5.7	4.4	2.3	3.5	5.0	5.8	4.7	0.2	0.2	1.3	4.3	1.3	2.1	4.8	5.2
Thaumarchaeota	0.4	1.6	1.4	7.4	8.1	13.1	0.8	2.3	6.5	10.9	12.0	0.4	0.1	0.8	9.7	0.5	0.5	3.2	6.4
Cyanobacteria	18.4	16.4	16.1	0.5	0.1	0.1	15.7	9.7	0.3	0.1	0.1	30.7	36.8	1.9	0.5	18.2	13.3	4.7	0.7
Alphaproteobacteria	39.4	26.2	37.1	25.4	22.3	17.1	39.4	38.4	27.8	22.9	20.0	22.8	22.4	35.2	31.7	37.2	43.7	31.9	25.2
Gammaproteobacteria	10.9	8.8	10.6	11.3	14.6	15.5	11.2	12.2	11.5	15.9	15.3	11.3	12.0	27.7	16.8	12.0	13.8	12.9	11.5
Deltaproteobacteria	0.7	0.9	1.3	11.1	15.2	19.2	0.8	1.3	9.5	14.7	18.5	0.4	0.4	0.6	7.0	1.0	1.5	6.7	12.1
Bacteroidetes	8.7	19.9	11.2	2.0	1.0	0.8	10.1	9.8	2.3	0.8	1.2	13.9	14.5	20.3	6.1	12.3	8.1	4.0	1.7
Actinobacteria	10.9	16.1	10.8	2.9	1.8	2.4	7.7	8.0	2.3	1.7	2.1	14.7	9.5	6.6	4.1	9.3	7.8	3.1	2.4
Acidobacteria	<0.01	0.1	0.1	1.7	1.1	1.3	<0.01	0.1	1.5	0.9	1.2	<0.01	<0.01	0.1	1.0	<0.01	<0.01	0.6	1.3
Chloroflexi	0.7	0.7	1.0	12.6	10.7	8.1	0.6	1.1	12.8	8.0	6.5	0.1	0.1	0.1	4.3	0.4	0.6	9.2	11.9
Firmicutes	<0.01	<0.01	<0.01	<0.01	<0.01	0.02	<0.01	<0.01	<0.01	<0.01	0.1	<0.01	<0.01	<0.01	<0.01	<0.01	<0.01	<0.01	0.2
Marinimicrobia (SAR406)	3.9	3.2	3.4	9.7	13.1	12.2	4.9	6.1	9.3	12.5	12.4	1.5	1.6	2.8	6.4	3.7	4.7	8.9	11.5
Nitrospinae	<0.01	<0.01	<0.01	0.4	0.4	0.3	<0.01	<0.01	0.3	0.4	0.3	<0.01	<0.01	<0.01	0.6	<0.01	<0.01	0.2	0.3
Nitrospirae	<0.01	<0.01	<0.01	0.1	0.3	<0.01	<0.01	<0.01	0.1	0.2	0.0	<0.01	<0.01	<0.01	<0.01	<0.01	<0.01	0.0	0.1
Planctomycetes	0.2	0.9	0.8	2.3	1.7	1.6	0.3	0.8	2.1	1.4	1.7	0.5	0.4	0.4	2.7	0.3	0.3	1.5	1.9
Gemmatimonadetes	<0.01	0.2	0.2	1.6	0.9	0.9	0.1	0.5	1.6	0.8	1.0	0.1	0.0	0.1	0.8	0.1	0.1	0.9	1.3
Verrucomicrobia	0.6	1.1	1.2	0.7	0.7	0.9	0.6	1.2	0.8	0.9	1.0	1.1	0.5	0.5	1.2	0.5	0.8	1.2	0.6
Others	4.0	3.3	3.5	5.8	2.6	2.1	5.3	4.9	6.3	2.3	1.9	2.2	1.7	1.6	2.8	3.3	2.6	6.2	5.8
OUT: >97% similarity																			

Table S5. Information about metagenomic and 16S rRNA gene amplicon sequences in this study

Metagenomic samples	St. 0						St. 1					St. 4				St. 5			
	0 m	40 m (SCM)	100 m	200 m	500 m	800 m	50 m (SCM)	100 m	200 m	500 m	800 m	0 m	40 m (SCM)	100 m	200 m	0 m	40 m (SCM)	100 m	200 m
Quantitiy of metagenomic DNA (ng)	1075	1320	1605	680	172	63	1570	880	995	128	69	510	465	286	138	1625	1955	1550	900
Volume of sequence data (GB)	7.9	6.1	5.5	7.0	7.3	7.4	7.9	8.8	6.9	6.8	6.5	8.7	8.9	6.5	7.2	8.5	7.6	6.3	6.5
No. of total reads	28339374	21888550	19867714	25244368	26277182	26355546	28334504	31487596	24732522	24023626	23275794	31146316	31822892	23256754	25670706	30249886	26808868	22308748	23078000
Counting *hgcAB* genes																			
No. of paired-end sequences after *quality check (>300 bp)	11200036	4893125	5132031	9341901	10245371	10302013	6739992	9226933	7235628	6912774	7051998	13498040	12701393	10204846	9932838	5971912	7013881	5936543	6820772
No. of predicted genes (>30 amino acids)	12176151	5392907	5680268	10253173	10556664	10853796	7686917	10289321	7508770	6894392	7177796	15673455	14997925	11738982	11178054	6103817	7447199	6156855	6889240
No. of *recA* sequences	10774	3273	3714	5609	4775	4988	4921	5980	3858	3084	3130	10218	9854	7348	5495	5076	5119	3173	3127
No. of *hgcA* sequences	0	0	0	0	7	21	0	0	0	13	21	0	0	0	0	0	0	0	0
No. of *hgcB* sequences	6	2	1	36	25	30	1	6	19	15	12	7	3	8	12	6	2	8	15
Phylogenetic analysis for *hgcAB* genes																			
No. of contigs after MEGAHIT assmbly**	1545291	1253469	1175454	1820883	1957310	1765617	1719186	1909447	1968085	1815152	1604335	1744922	1660259	1196184	1724079	1783244	1695616	1711090	1810173
N50 of contigs (bp)	588	519	536	655	554	551	507	558	557	501	518	592	621	618	620	477	506	547	550
Minimum length of contigs (bp)	200	200	200	200	200	200	200	200	200	200	200	200	200	200	200	200	200	200	200
Average length of contigs (bp)	587.2	523.6	534.5	642.5	550	546.7	513	558	559.2	499.5	515.2	583.9	606.3	603.2	612.8	492.4	509.6	548.4	548.2
Maximum length of contigs (bp)	49381	41671	38043	86934	31685	16929	13224	43898	75278	26146	18332	36593	57463	238305	51188	29725	64726	44536	24056
No. of predicted genes (>30 amino acids)	1877315	1450467	1401074	2337365	2236410	2028211	2116352	2395394	2364328	1982190	1798329	2273269	2184223	1544515	2237925	1870378	1936418	2036712	2115861
Mapped reads	18997663	12696624	11524265	14757326	13997675	15384262	18000463	23134168	15407780	14091403	14689018	22637977	23905167	17816074	14682129	19398981	17350839	13551044	12587174
Coverage of mapped read (percentage of total reads)	67.0	58.0	58.0	58.5	53.3	58.4	63.5	73.5	62.3	58.7	63.1	72.7	75.1	76.6	57.2	64.1	64.7	60.7	54.5
No. of *hgcA* sequences	0	0	0	0	1	3	0	0	0	1	3	0	0	0	0	0	0	0	0
No. of *hgcB* sequences	7	4	3	30	13	15	4	17	24	10	12	7	7	2	14	6	4	19	15
16S rRNA amplicon sequence reads	40302	55179	46592	48875	47342	41193	47887	50093	51844	42782	43351	47273	42356	38011	41709	39525	50024	46278	45537

*Remove the low quality sequences after paired-end assembly

**Assemble condition (k-min = 21, k-max = 141, and k-step = 12)

Table S6. *hgcA*-like sequences for phylogenetic analysis. Bold letters in sequences indicated the cap-helix.

Contig name	*hgcA* sequence detected in this study (amino acid sequence)
St0_500m_contig_568431	MINWIKDIYQTLFRFARFPCEPETVTIGNPDKSSPVLVTCNFDYTVRHLKEYLKKEALDCFLLVVNTKGT**NVWCAATEG**VFTTDIVLSHLKVYNVGELVNHKRLILPQLSVAGVKRKELKEHGWEGIYGPVYFTDLKEFLNNGLTKNKDMQALEYGYWERFKMGLSHAVFCTLVCIIPIFLFASDWWIQGIGLVWYFAFSMQLIEHFIPFERLLYKGLALSLPILILTLTSITDPVLKTQATLGVIALGGYIGYDAQGHSHLGQNQKSGTIFAGMFAFLALVYGGTLFL
St0_800m_contig_424282	MIDWIKDIYQTLFRFARFPCEPDTVLIGNPDKSSPVLVTCNFDYTVRHLKDYLEKEQLDCFLLVVNTKGT**NVWCAAAEG**IFTTDTVLSHLKVYNVGELVNHHQLILPQLSVAGVKRKELKEHGWEGIYGPVYFADLKEFLNNGLTKNKDMQALEYGYWERFKMGLSHAVFCTLVCIVPIFLFASDYWIQAIVLVWYFAFCMQLIEHFIPFTRLLYKGLVLSLPILALALTSVKDPVLKIQVTMGIIALGAYIGYDA
St0_800m_contig_1380379	MINWIKDIYQTLFRFARFPCEPETVTIGNPDKSSPVLVTCNFDYTVRHLKDYLKKEALDCFLLVVNTKGT**NVWCAAAEG**VFTTDIVLSHLKVYNVGELVNHKRLILPQLSVAGVKRKELKEHGWEGIYGPVYFTDLKEFLNNGLTKNKDMQALEYGYWERFKMGLSHAVFCTLVCIIPIFLFASDWWIQGIGLVWYFAFNMQLIEHFIPFERLLYKGLALSLPILVLTLTSITDPVLKTQATLGVIALGGYIGYDAQGHSHLGQNQKSGAIFAGMFSFLALVYGGTLFL
St0_800m_contig_1480338	MIGWIKDIYQTLFRFARFPCEPETIAIGSPDKSSPILVTCNFDYTVRHLKEYLKKEALDCFLLVVNTKGT**NVWCAAAEG**VFTTDTVLSHLKVYNVGELVNHKRLILPQLSVAGVKRKELKEHGWEGIYSPVYITDLKEFLNNGLTKNKDMQALEYDYWERFEIGLSHAVFCTLICIIPIFLFTSDWWIQGIGLVWYFA
St1_500m_contig_646140	MINWIKDIYQTLFRFARFPCEPETVTIGNPDKSSPVLVTCNFDYTVRHLKEYLKKEALDCFLLVVNTKGT**NVWCAAAEG**VFTTDIVLSHLKVYNVGELVNHKRLILPQLSVAGVKRKELKEHGWEGIYGPVYFTDLKEFLNNGLTKNKDMQALEYGYWERFKMGLSHAVFCTLVCIIPIFLFASDWWIQGIGLVWYFAFSMQLIEHFIPFERLLYKGLALSLPILVLTLTSITDPVLKTQATLGVIALGGYIGYDAQGHSHLGQNQKSGTIFAGMFAFLALVYGGTLFL
St1_800m_contig_127486	MIAWVKDIYQTLFRFARFPCEPETVAVGNPDKSSPVLVTCNFDYTVRHLKKYLEKEQLDCFLLVVNTKGT**NVWCAAAEG**IFTTETVLAHLKVYNVKDLVDHTRLILPQLSVAGIKRKDLKEHGWEGIYGPVYFTDLKEFLKNGLTKTKEMQALEYGYWERFKMGLSHAVFCTLVCILPIFLFASDWWLQAIALVWYFAFSMQLIEHFIPFHRLLYKGLALTLPVLAIALFSVTDSVLRIQATIGIVALGAYIGYDAQGHSHLGQNQQSGKLFARIFASLALIYGGTLLL
St1_800m_contig_783837	MINWIKDIYQTLFRFARFPCEPETVTIGNPDKSSPVLVTCNFDYTVRNLKEYLKKEALDCFLLVVNTKGT**NVWCAAAEG**VFTTDIVLSHLKVYNVGELVNHKRLILPQLSVAGVKRKELKEHGWEGIYGPVYFTDLKEFLNNGLTKNKDMQALEYGYWERFKMGLSHAVFCTLVCII
St1_800m_contig_783838	MIGWIKDIYQTLFRFARFPCEPETIAIGSPDKSSPILVTCNFDYTVRHLKEYLKKEALDCFLLVVNTKGT**NVWCAAAEG**VFTTDTVLSHLKVYNVGELVNHKRLILPQLSVAGVKRKELKEHGWKGIYGPVYFTDLKEFLNNGLTKNKDMQALEYGYWERFKMGLSHAVFCTLVCII

Table S7. Raw data of the MAPLE analysis (total module abundance in small category after whole community analysis).

Small category	Abundance of modules (arbitrary unit)																	
	St0_0m	St0_SCM	St0_100m	St0_200m	St0_500m	St0_800m	St1_SCM	St1_100m	St1_200m	St1_500m	St1_800m	St4_0m	St4_SCM	St4_100m	St4_200m	St5_0m	St5_SCM	St5_100m	St5_200m
ABC-2 type and other transport systems	2.283	3.348	3.332	4.225	4.565	4.309	3.3	3.703	4.452	4.364	4.36	3.612	3.561	4.107	4.186	2.452	3.271	4.212	4.566
Aminoacyl tRNA	0.628	0.987	0.977	1.278	1.36	1.341	1.067	1.154	1.398	1.377	1.383	1.002	0.973	1.109	1.267	0.742	0.961	1.295	1.368
Arginine and proline metabolism	1.252	2.375	2.43	2.47	2.563	2.336	2.674	2.499	2.644	2.399	2.388	2.645	2.597	2.561	2.207	1.324	2.477	2.619	2.58
Aromatic amino acid metabolism	0.991	1.875	1.832	1.816	1.936	1.771	1.84	2.003	1.935	1.838	1.811	2.061	2.064	2.241	1.831	1.068	1.71	2.077	1.951
Aromatics degradation	0.025	0.019	0.019	0.077	0.079	0.05	0.013	0.027	0.071	0.056	0.045	0.015	0.015	0.016	0.044	0.021	0.018	0.107	0.068
ATP synthesis	3.78	3.638	3.652	3.499	3.302	3.174	3.845	3.806	3.373	3.321	3.319	3.722	3.665	3.905	3.467	3.882	3.962	3.541	3.402
Bacterial secretion system	0.833	1.606	1.647	1.449	1.427	1.462	1.567	1.495	1.369	1.383	1.335	1.71	1.719	1.639	1.469	0.893	1.612	1.447	1.343
Branched-chain amino acid metabolism	2.918	2.617	2.857	3.488	3.5	3.366	3.2	2.974	3.694	3.714	3.462	2.791	2.937	3.078	3.268	3.193	3.3	3.461	3.69
Carbon fixation	2.442	1.698	2.018	1.073	1.076	1.134	2.344	1.709	1.144	1.182	1.228	2.084	1.98	1.337	1.188	2.357	2.315	1.577	1.097
Central carbohydrate metabolism	5.383	5.094	5.433	5.978	6.353	6.009	5.977	5.55	6.376	6.243	6.22	5.451	5.56	5.472	5.595	5.781	6.079	6.348	6.6
Cofactor and vitamin biosynthesis	2.346	2.335	2.471	2.645	3.178	3.021	2.659	2.613	2.694	3.121	3.062	2.615	2.796	3.405	2.708	2.505	2.632	3.065	2.847
Cysteine and methionine metabolism	1.301	0.897	1.084	0.71	0.859	0.726	1.313	1.01	0.832	0.823	0.837	1.165	1.156	1.12	0.811	1.266	1.289	1.007	0.795
DNA polymerase	0.169	0.569	0.535	0.433	0.394	0.342	0.502	0.547	0.45	0.382	0.389	0.673	0.617	0.579	0.531	0.213	0.415	0.534	0.444
Drug efflux transporter/pump	0.252	0.281	0.305	0.194	0.202	0.168	0.363	0.289	0.224	0.185	0.175	0.324	0.344	0.319	0.214	0.288	0.337	0.274	0.219
Drug resistance	0.92	0.759	0.757	1.119	1.185	1.05	0.835	0.85	1.166	1.166	1.126	0.681	0.766	1.165	0.89	0.924	0.839	1.12	1.151
Fatty acid metabolism	4.318	3.535	3.513	4.197	4.377	4.038	4.016	3.995	4.288	4.266	4.277	3.667	3.837	4.42	3.879	4.664	3.86	4.379	4.551
Glycosaminoglycan metabolism	0.002	0.002	0.004	0.005	0.016	0.008	0.001	0.002	0.01	0.011	0.016	0.003	0.003	0.005	0.008	0.003	0.003	0.009	0.01
Histidine metabolism	0.051	0.062	0.078	0.217	0.265	0.223	0.054	0.073	0.217	0.278	0.251	0.063	0.079	0.141	0.205	0.057	0.042	0.231	0.225
Lipid metabolism	0.239	0.461	0.464	0.501	0.444	0.414	0.447	0.505	0.489	0.449	0.454	0.487	0.443	0.404	0.452	0.239	0.453	0.499	0.515
Lipopolysaccharide metabolism	0.197	0.365	0.315	0.623	0.705	0.749	0.306	0.35	0.626	0.745	0.814	0.283	0.323	0.552	0.567	0.241	0.298	0.529	0.641
Lysine metabolism	0.859	0.929	1.012	0.978	0.903	0.873	1.134	1.009	1.023	0.985	0.903	1.017	1.046	1.045	1.004	0.909	1.144	1.007	1.044
Metabolic capacity	0.132	0.056	0.077	0.019	0.028	0.035	0.087	0.05	0.022	0.031	0.049	0.108	0.108	0.005	0.02	0.124	0.088	0.058	0.03
Metallic cation, iron-siderophore and vitamin B12 transport system	1.091	0.816	0.995	0.875	0.532	0.405	1.158	0.864	0.93	0.467	0.44	1.034	1.009	0.71	0.812	1.018	1.196	1.01	0.931
Methane metabolism	0.049	0.055	0.045	0.245	0.392	0.422	0.04	0.078	0.285	0.371	0.341	0.027	0.031	0.062	0.237	0.04	0.03	0.17	0.337
Mineral and organic ion transport system	1.827	2.346	2.355	2.776	3.476	3.246	2.57	2.516	2.967	3.484	3.375	2.29	2.368	3.074	2.534	2.057	2.439	2.634	2.968
Nitrogen metabolism	0.075	0.054	0.075	0.031	0.027	0.034	0.07	0.042	0.021	0.024	0.045	0.088	0.091	0.005	0.032	0.074	0.077	0.053	0.047
Nucleotide sugar	0.057	0.071	0.065	0.218	0.247	0.333	0.064	0.091	0.243	0.298	0.369	0.041	0.063	0.091	0.269	0.074	0.066	0.185	0.264
Other amino acid metabolism	0.01	0.009	0.008	0.018	0.012	0.013	0.007	0.009	0.013	0.019	0.011	0.004	0.007	0.005	0.024	0.007	0.01	0.022	0.02
Other carbohydrate metabolism	1.289	1.226	1.194	1.511	1.747	1.843	1.326	1.342	1.606	1.757	1.88	1.213	1.33	1.461	1.491	1.326	1.273	1.491	1.549
Pathogenicity	0	0	0	0	0.002	0	0	0	0	0	0.001	0	0	0	0.001	0	0	0.002	0
Peptide and nickel transport system	0.553	0.636	0.632	0.764	0.917	0.824	0.713	0.743	0.829	0.913	0.858	0.639	0.704	0.876	0.729	0.676	0.655	0.794	0.858
Phosphate and amino acid transport system	2.789	2.621	2.827	2.952	3.182	2.864	3.213	2.892	3.076	3.103	3.086	2.739	2.722	2.88	2.51	3.001	3.363	3.117	3.088
Phosphotransferase system (PTS)	0.079	0.063	0.053	0.018	0.007	0.008	0.03	0.039	0.023	0.011	0.01	0.055	0.039	0.037	0.046	0.082	0.048	0.022	0.018
Photosynthesis	0.508	0.275	0.408	0.009	0	0	0.47	0.256	0.009	0	0	0.484	0.427	0.05	0.021	0.589	0.592	0.171	0.015
Polyamine biosynthesis	0.165	0.256	0.334	0.33	0.297	0.214	0.352	0.247	0.276	0.283	0.248	0.403	0.337	0.13	0.222	0.257	0.374	0.339	0.345
Proteasome	0.066	0.042	0.053	0.096	0.17	0.127	0.058	0.051	0.096	0.143	0.137	0.031	0.034	0.038	0.065	0.066	0.045	0.086	0.113
Purine metabolism	1.543	2.609	2.558	1.88	1.909	1.827	2.814	2.035	2.029	2.24	1.866	2.671	2.636	2.702	1.876	1.636	2.588	1.952	1.984
Pyrimidine metabolism	1.157	2.076	1.962	2.252	2.421	2.461	1.94	2.135	2.306	2.557	2.597	2.056	2.053	2.133	2.35	1.229	1.94	2.11	2.323
Ribosome	0.286	0.61	0.552	0.361	0.269	0.179	0.483	0.66	0.481	0.226	0.186	0.609	0.635	0.931	0.419	0.383	0.418	0.533	0.357
RNA polymerase	1.094	1.007	1.043	0.995	0.913	0.869	1.279	1.091	0.892	0.809	0.83	1	0.992	1.024	0.941	1.101	1.143	1.114	0.882
RNA processing	0.041	0.033	0.04	0.179	0.274	0.334	0.046	0.057	0.192	0.326	0.287	0.028	0.025	0.059	0.254	0.032	0.03	0.125	0.212
Saccharide, polyol, and lipid transport system	2.086	1.824	1.923	1.951	1.825	1.598	2.2	2.163	2.043	1.702	1.675	1.831	2.087	3.015	1.992	2.316	2.209	2.282	1.854
Serine and threonine metabolism	0.789	1.065	1.04	1.214	1.353	1.416	1.061	1.109	1.272	1.486	1.518	1.142	1.137	1.281	1.252	0.9	1.033	1.214	1.298
Sulfur metabolism	0.191	0.197	0.262	0.345	0.417	0.511	0.293	0.198	0.358	0.543	0.54	0.283	0.281	0.152	0.428	0.192	0.346	0.343	0.391
Terpenoid backbone biosynthesis	0.232	0.263	0.197	0.639	0.768	0.965	0.225	0.373	0.713	0.992	0.993	0.195	0.23	0.482	0.939	0.269	0.206	0.545	0.79
Two-component regulatory system	1.045	2.724	2.522	2.137	1.727	1.688	2.669	2.88	2.207	1.667	1.74	2.732	2.664	2.952	2.181	1.335	2.452	2.368	2.079
